# Attractor-Like Dynamics Extracted from Human Electrocorticographic Recordings Underlie Computational Principles of Auditory Bistable Perception

**DOI:** 10.1523/JNEUROSCI.1531-22.2023

**Published:** 2023-05-03

**Authors:** Pake Melland, Rodica Curtu

**Affiliations:** ^1^Department of Mathematics, Southern Methodist University, Dallas, Texas 75275; ^2^Applied Mathematical & Computational Sciences, The University of Iowa, Iowa City, Iowa 52242; ^3^Department of Mathematics, The University of Iowa, Iowa City, Iowa 52242; ^4^The Iowa Neuroscience Institute, The University of Iowa, Iowa City, Iowa 52242

**Keywords:** attractors, bistable perception, embeddings, feature extraction, neural manifold

## Abstract

In bistable perception, observers experience alternations between two interpretations of an unchanging stimulus. Neurophysiological studies of bistable perception typically partition neural measurements into stimulus-based epochs and assess neuronal differences between epochs based on subjects' perceptual reports. Computational studies replicate statistical properties of percept durations with modeling principles like competitive attractors or Bayesian inference. However, bridging neuro-behavioral findings with modeling theory requires the analysis of single-trial dynamic data. Here, we propose an algorithm for extracting nonstationary timeseries features from single-trial electrocorticography (ECoG) data. We applied the proposed algorithm to 5-min ECoG recordings from human primary auditory cortex obtained during perceptual alternations in an auditory triplet streaming task (six subjects: four male, two female). We report two ensembles of emergent neuronal features in all trial blocks. One ensemble consists of periodic functions that encode a stereotypical response to the stimulus. The other comprises more transient features and encodes dynamics associated with bistable perception at multiple time scales: minutes (within-trial alternations), seconds (duration of individual percepts), and milliseconds (switches between percepts). Within the second ensemble, we identified a slowly drifting rhythm that correlates with the perceptual states and several oscillators with phase shifts near perceptual switches. Projections of single-trial ECoG data onto these features establish low-dimensional attractor-like geometric structures invariant across subjects and stimulus types. These findings provide supporting neural evidence for computational models with oscillatory-driven attractor-based principles. The feature extraction techniques described here generalize across recording modality and are appropriate when hypothesized low-dimensional dynamics characterize an underlying neural system.

**SIGNIFICANCE STATEMENT** Irrespective of the sensory modality, neurophysiological studies of multistable perception have typically investigated events time-locked to the perceptual switching rather than the time course of the perceptual states per se. Here, we propose an algorithm that extracts neuronal features of bistable auditory perception from largescale single-trial data while remaining agnostic to the subject's perceptual reports. The algorithm captures the dynamics of perception at multiple timescales, minutes (within-trial alternations), seconds (durations of individual percepts), and milliseconds (timing of switches), and distinguishes attributes of neural encoding of the stimulus from those encoding the perceptual states. Finally, our analysis identifies a set of latent variables that exhibit alternating dynamics along a low-dimensional manifold, similar to trajectories in attractor-based models for perceptual bistability.

## Introduction

Multistable perception, a phenomenon in which an ambiguous, unchanging stimulus gives rise to more than one perceptual interpretation, has been found in various sensory modalities: visual (Blake, 1989; Hupé and Rubin, 2003), auditory (van Noorden, 1975; Pressnitzer and Hupé, 2006), tactile (Carter et al., 2008), and olfactory (Zhou and Chen, 2009). Visual and auditory research has also reported neural correlates to mutually exclusive percepts (Blake and Logothetis, 2002; Micheyl et al., 2007), proposing several theories of bistable perceptual organization. Nonetheless, computational principles of bistable perception are yet to be reconciled with experimentally identified percept-specific changes in neural activity. This is because modeling simulates long-time neuronal dynamics during single trials with functional principles such as competitive attractors (Moreno-Bote et al., 2007; Curtu et al., 2008; Rankin et al., 2015), evidence accumulation (Barniv and Nelken, 2015; Nguyen et al., 2020), algorithmic signal detection (Micheyl et al., 2005; Pressnitzer et al., 2008; Krishnan et al., 2014), predictive coding (Denham and Winkler, 2006), or probabilistic processes (Barniv and Nelken, 2015). In contrast, conventional data analyses primarily rely on statistical measures within perceptual groupings, like mean and variance, and are thus limited when applied to nonstationary data. Accordingly, brain studies of bistable perception have focused on differentiating short-time neural responses near perceptual switches (Basirat et al., 2008; Higgins et al., 2020) or at fixed latencies in stimulus-locked epochs, like milliseconds from the onset of individual stimuli occurrences (Gutschalk et al., 2005; Snyder et al., 2006; Dykstra et al., 2011; Hill et al., 2012; Billig et al., 2018; Curtu et al., 2019; Higgins et al., 2020).

To unravel dynamical properties of neural activity in bistable perception and link them to functional principles proposed by theory and modeling, one must exploit the time dependency of the recorded data. Key components of a comprehensive analysis should include extraction of neural features with data-driven algorithms agnostic to the behavioral data (as opposed to prescribed measures such as averaged evoked potential) and identification of feature attributes that correlate with perception over prolonged percept durations as well as near reported switches or in other short-time windows. Here, we propose an algorithm that successfully addresses both problems. It inputs single-trial, minutes-long recordings of neuronal activity and outputs a manifold built on features that distinguish the two perceptual states. The algorithm predicts within-trial ongoing perceptual alternations (admittedly, without uncovering their neural underpinnings) by extracting time-varying latent states from neural activity compatible with trajectories in competition models for bistable perception.

**Figure 1.**
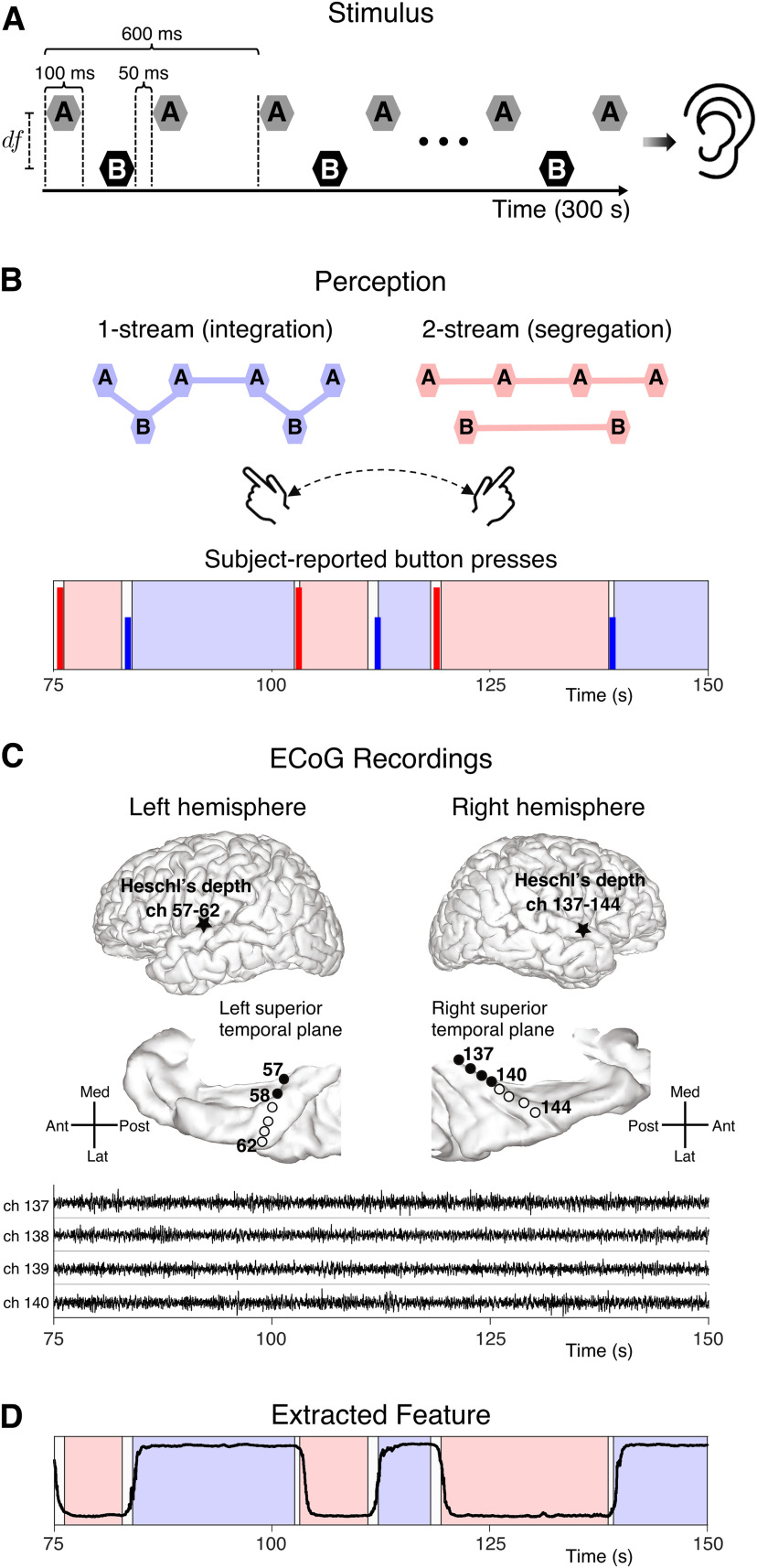
Overview of auditory triplet streaming task and recorded data. ***A***, The auditory stimulus was a sequence of high (***A***) and low (***B***) frequency tones separated by df semitones. The stimulus contained 500 *ABA*– triplets giving a total duration of 300 s. ***B***, Throughout the task, subjects reported alternations in perception by pressing buttons in a response box. Subject B335 button presses in response to control stimuli are shown from a 75-s window. Tall red (short blue) vertical lines indicate the onset of two-stream (one-stream) perception. ***C***, Electrocorticography (ECoG) recordings were simultaneously recorded during the behavioral task. Here, we examine only electrodes from posteromedial Heschl's gyrus (HGPM). Recordings from four HGPM contacts are shown over the same 75 s as in panel ***B*** (contacts placed in the right hemisphere of B335). ***D***, An example of an extracted neural feature derived from ECoG recordings alone. Features are timeseries equal in length to the duration of the stimulus. The background is shaded according to the subject-reported perception in panel ***B***, for comparison purposes.

Our study examined the dynamics of bistable perception in auditory streaming of triplets. Six neurosurgical patients listened to a sequence of tones, *A* and *B*, organized in repeating *ABA*– triplet patterns (Fig. 1). Subjects reported alternations between two percepts, a galloping-like rhythm (the “one-stream” percept) and a Morse-code-like rhythm of two simultaneous distinct streams (the “two-stream” percept). Electrocorticography (ECoG) recordings were collected from the subjects' core auditory cortex as they performed the behavioral task. Nonstationary percept-related features of the ECoG data were extracted with an algorithm built on recent advances in dynamical systems (Schmid, 2010; Williams et al., 2015; Giannakis, 2019), manifold learning (Takens, 1981; Berry et al., 2013), and dimensionality reduction of large-scale datasets (Coifman and Lafon, 2006; Nadler et al., 2006; Berry et al., 2013). The dynamics of neural activity were analyzed at multiple time scales: minutes, for the within-trial alternating process; seconds, for durations of individual percepts; and milliseconds, for the timing of switches. A collection of data-driven Fourier-like neuronal components robustly constructed the triplet-based averaged auditory evoked potential for the one-stream and two-stream percepts. An additional slowly-evolving extracted rhythm correlated with the perceptual states. Changes in the phase (rather than amplitude) of an oscillatory feature time-locked to the slow rhythm predicted the perceptual switches identified by subject-reported button presses. Low-dimensional projections of single-trial ECoG auditory cortical data revealed geometric structures common across subjects and stimulus types. These projections exhibited dynamic properties similar to trajectories generated by attractor-based computational models.

## Results

Six epileptic neurosurgical patients (identified here as B335, L357, R369, L372, R376, and L409), listened to 5-min-long sequences of tones *A* and *B* grouped in 500 triplets *ABA*–, each of 600ms duration (Fig. 1*A*). Participants indicated changes in perception by pressing a button on a response box. They reported either a single coherent auditory stream (the one-stream percept, ABA−ABA−...), or two simultaneous distinct streams (the two-stream percept, *A*–*A*–*A*–*A*–... and –*B*–*B*–...; Fig. 1*B*). Intracranial ECoG recordings from core auditory cortex were obtained concurrently with the behavioral data. The recordings were obtained from electrodes placed in posteromedial Heschl's gyrus (HGPM), with a total number of six (B335), five (L357), eight (R369), six (L372), seven (R376), and one (L409) contacts (Fig. 1*C* for B335).

Two stimulus protocols were employed. In the control block, df2-12, the stimulus consisted of several triplets alternating between low (*df* = 2) and high (*df* = 12) semitone difference between tones *A* and *B*, that biased listeners toward stable one-stream and two-stream percepts, respectively. Changes in perception were primarily aligned with the changes in df (Curtu et al., 2019). In the bistable blocks *df*6, *df*8, the stimulus consisted of triplets with fixed semitone difference between *A* and *B* throughout the entire task (either *df* = 6, or *df* = 8). Although the stimulus did not change, the subjects reported spontaneous alternations between one-stream and two-stream percepts.

The datasets analyzed in this paper were previously published by Curtu et al. (2019).

### An algorithm for extracting nonstationary features from ECoG data

Studies of perceptual bistability aim to identify changes in neural activity that correlate with changes in (simultaneously recorded) behavioral responses. In auditory streaming of triplets, several studies have reported differences in the averaged auditory evoked potential calculated for the one-stream and two-stream percepts, in certain auditory-related brain areas. But these analyses used a common methodology: (1) obtained high-resolution temporal recordings with either electroencephalography (EEG; Hill et al., 2012), magnetoencephalography (MEG; Gutschalk et al., 2005; Billig et al., 2018), or ECoG (Curtu et al., 2019); (2) partitioned the data in triplet-locked epochs; then (3) performed univariate or multivariate statistics over triplets belonging to each percept. More recently, nonlinear measures derived from entropy principles were also used to identify neuronal differences between the percepts (Canales-Johnson et al., 2020), but they were based on short-time windows near switches and were reliant on the subjects' behavioral reports. We present here an algorithm that successfully processes minutes-long nonstationary neural data (as seen in the streaming task), and extracts key features across three different timescales. The algorithm identifies perceptual-related events at times measured in: milliseconds (the occurrence of switches between percepts), seconds (the sequence of triplets linked into a stable percept, either one-stream or two-stream), and minutes (the perceptual dynamics over the entire 5-min auditory block). Moreover, this algorithm, was applied exclusively to the neural data without using any prior knowledge about the perception.

The fundamental assumption guiding this feature-extraction algorithm is that a potentially nonlinear system of differential equations,

(1) $$\frac{\text{d}x}{\text{d}t}=f(x),\;x\in {\mathbb{R}}^{n}$$

governs the internal brain response, *x*, to auditory input (here we consider that the brain activity could be described by the dynamics of *n* variables). We further assume that a time-dependent trajectory x(t) which satisfies Equation 1 evolves along a lower dimensional manifold (say, of dimension *d*) $M\subseteq {\mathbb{R}}^{n}$. Several core mechanisms in the peripheral and central auditory system influence the dynamics in Equation 1. Therefore, realizations of Equation 1 are challenging to observe directly. Instead, we rely on observations of the system, y=g(x), where $y\in {\mathbb{R}}^{n_{c}}$ represents a measurable quantity, and g(⋅) is a vector of smooth, yet still unknown, scalar functions of the underlying brain state *x*. In the streaming context, local field potential (LFP) recordings from HGPM contacts represent the output of unknown observation functions of the unknown underlying neural activity. Thus, the number of HGPM contacts, $n_{c}$, determines the observation dimension.

We hypothesize that the manifold M contains regions, or almost-invariant attracting sets, that correspond with perception (S.L. Brunton et al., 2017; also, for the terminology, see Materials and Methods). Under this hypothesis, a trajectory x(t) is contained in an almost-invariant space over the duration of a percept. Then, near a perceptual switch, it transitions to another attracting set. However, the underlying manifold M and the true dynamics are likely obfuscated by the observation functions, so one needs to invoke methods designed to extract intrinsic dynamical patterns from observational (ECoG) data.

Recently, the Koopman operator (Koopman, 1931; Koopman and Neumann, 1932; Mezić and Banaszuk, 2004; Mezić, 2005; Rowley et al., 2009; S.L. Brunton et al., 2017, 2022) has been used to study nonlinear dynamics through system observations, for example in fluid dynamics (Rowley et al., 2009; Mezić, 2013; Peitz and Klus, 2019), computational chemistry (Narasingam and Kwon, 2019), and neuroscience (B.W. Brunton et al., 2016; Cura and Akan, 2020; Marrouch et al., 2020). For time *t* fixed, the Koopman operator $K_{t}$ is defined by composing observation functions with the flow map (i.e., the solution) $F_{t}(x):M\to M$ for Equation 1:

$$(K_{t}g)(x)=g(F_{t}(x)).$$

In many scientific applications, observations are made at discrete time points $t_{k}$ sampled over a temporal interval Δt. The sampling procedure yields a sequence of state variables $x_{k}$, whose dynamics along M are determined by the discrete map, $x_{k+1}=F_{\Delta t}(x_{k})$. Hence, for an observation made at time $t_{k}$, given by $y_{k}=g(x_{k})$, the temporal evolution of the observation over Δt is given by $\left(K_{\Delta t}g)(x_{k})=g(F_{\Delta t}(x_{k}))=g(x_{k+1}\right)$. Thus, a proper characterization of the Koopman operator can describe the dynamics in system measurements or observations. An emerging goal in data science is to approximate K from a time series of observations.

By the definition of function composition, the Koopman operator is linear; however, since it acts on the functional space of system observations, it is infinite-dimensional. Rather than identify the full infinite dimensional operator, common approaches characterize the operator by approximating a finite collection of its leading eigenvalues, modes, and eigenfunctions $\left\{(\omega_{j},v_{j},\varphi_{j})\right\}$. This is typically accomplished by calculating spectral features of the finite matrix K that minimizes the squared residual error over all measurements with the prediction:

(2) $$y_{k+1}=Ky_{k}.$$

When the observation dimension is large, the dynamic mode decomposition (DMD; Rowley et al., 2009; Schmid, 2010; Tu et al., 2014) is an effective algorithm for approximating Koopman spectral quantities.

For the data presented in this paper, the number of HGPM contacts was subject dependent and varied between one and eight, yielding few observations relative to the total number of sampled time points. For low-dimensional observations, the extended dynamic mode decomposition (eDMD; Williams et al., 2015) augments the approximation in Equation 2 to act on features of the observation data. Since we were interested in the geometry of the system's underlying state space, we used manifold learning techniques to derive a collection of basis-like features (or functions) adapted to the geometry along M. These data-driven features served as a dictionary of functions for Koopman eigenfunction discovery with the eDMD algorithm.

**Figure 2.**
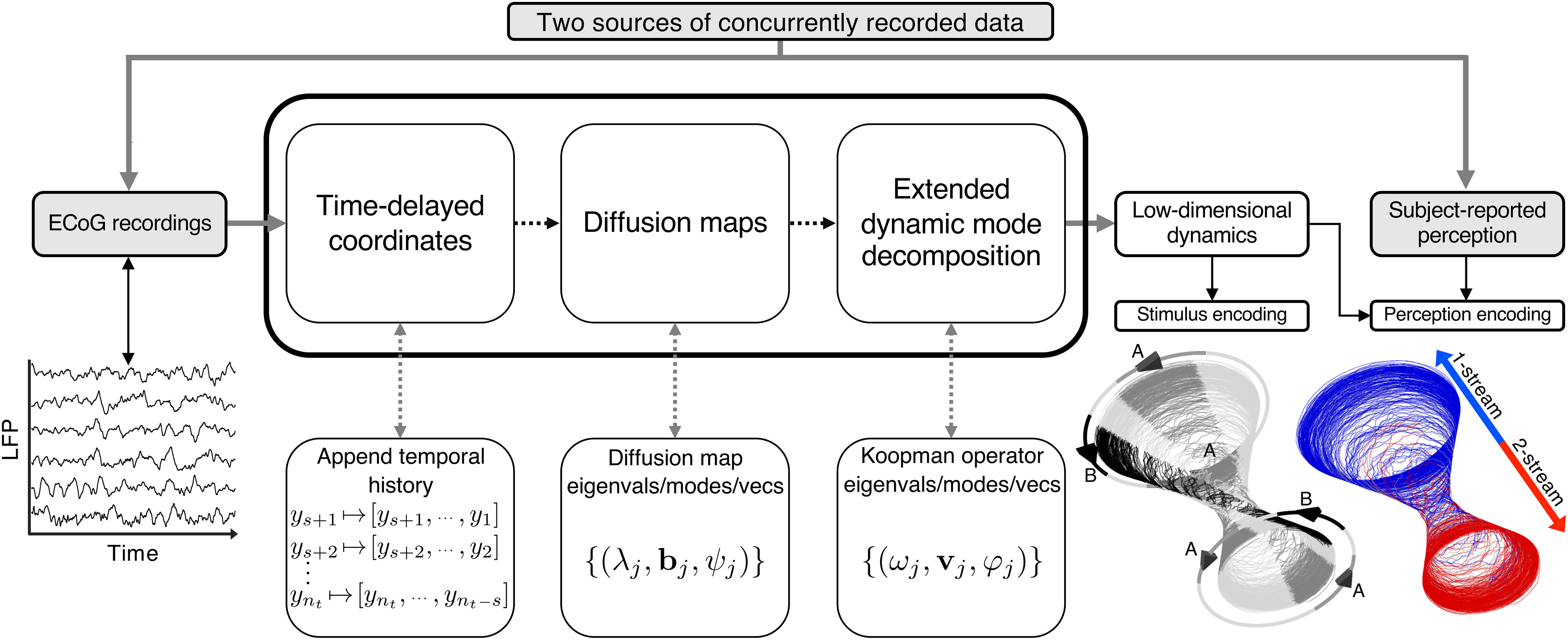
End-to-end computational approach for extracting low-dimensional dynamics from neural data. Single-subject electrocorticography (ECoG) data measuring local field potentials (LFPs) were input for a feature extraction algorithm that successively applied the methods of time-delayed coordinates (Takens, 1981; Sauer et al., 1991), diffusion maps (Coifman and Lafon, 2006; Berry et al., 2013), and the extended dynamic mode decomposition (Williams et al., 2015). The output is a low-dimensional collection of timeseries features, $\varphi_{j}$, that were studied for their connection to subject-reported percepts in auditory streaming of triplets. Shown here are three-dimensional representations of approximated Koopman eigenfunctions $\varphi_{j}$ (trajectories lying on an hourglass-like manifold) illustrating two key properties of the streaming task: stimulus encoding (repeated *ABA*– triplets; in gray) and perception encoding (one-stream vs two-stream percept; in blue vs red color).

To derive a function dictionary for the eDMD algorithm, we first employed time-delay coordinates (Takens, 1981; Sauer et al., 1991) to reconstruct and embed the state-space dynamics in a high-dimensional ambient space by appending temporal lags to a time series of ECoG recordings (Fig. 2). As suggested previously (Berry et al., 2013), we then applied the diffusion map algorithm (Coifman et al., 2005; Coifman and Lafon, 2006; Nadler et al., 2006) to the augmented high-dimensional data to provide low-dimensional timeseries representations, $\psi_{j}$, that preserve the state-space dynamics revealed with delay coordinates. From a theoretical perspective, the diffusion map features $\psi_{j}$ are eigenvectors of a stochastic matrix derived from the high-dimensional delay coordinates. The eigenvectors represent an approximation to a Fourier-like basis of square-integrable functions adapted to dynamics along the underlying manifold M (Coifman and Lafon, 2006; Berry et al., 2013; Giannakis, 2019). The diffusion map features were then used as a dictionary for the eDMD algorithm (Williams et al., 2015) to approximate Koopman eigenvalues, modes, and eigenfunctions $\left\{(\omega_{j},v_{j},\varphi_{j})\right\}$, which link the dynamics of the observations *y* to the dynamics of the unknown state *x*. Figure 2 depicts the end-to-end algorithm (for more details, see Materials and Methods).

The Koopman quantities were derived from ECoG data alone, independently of perception-reports, and were subsequently studied for characteristics relevant to the triplet streaming task. See Figure 1*D* for an exemplar feature which correlated with perception.

### Eigenvalues organize into two branches

**Figure 3.**
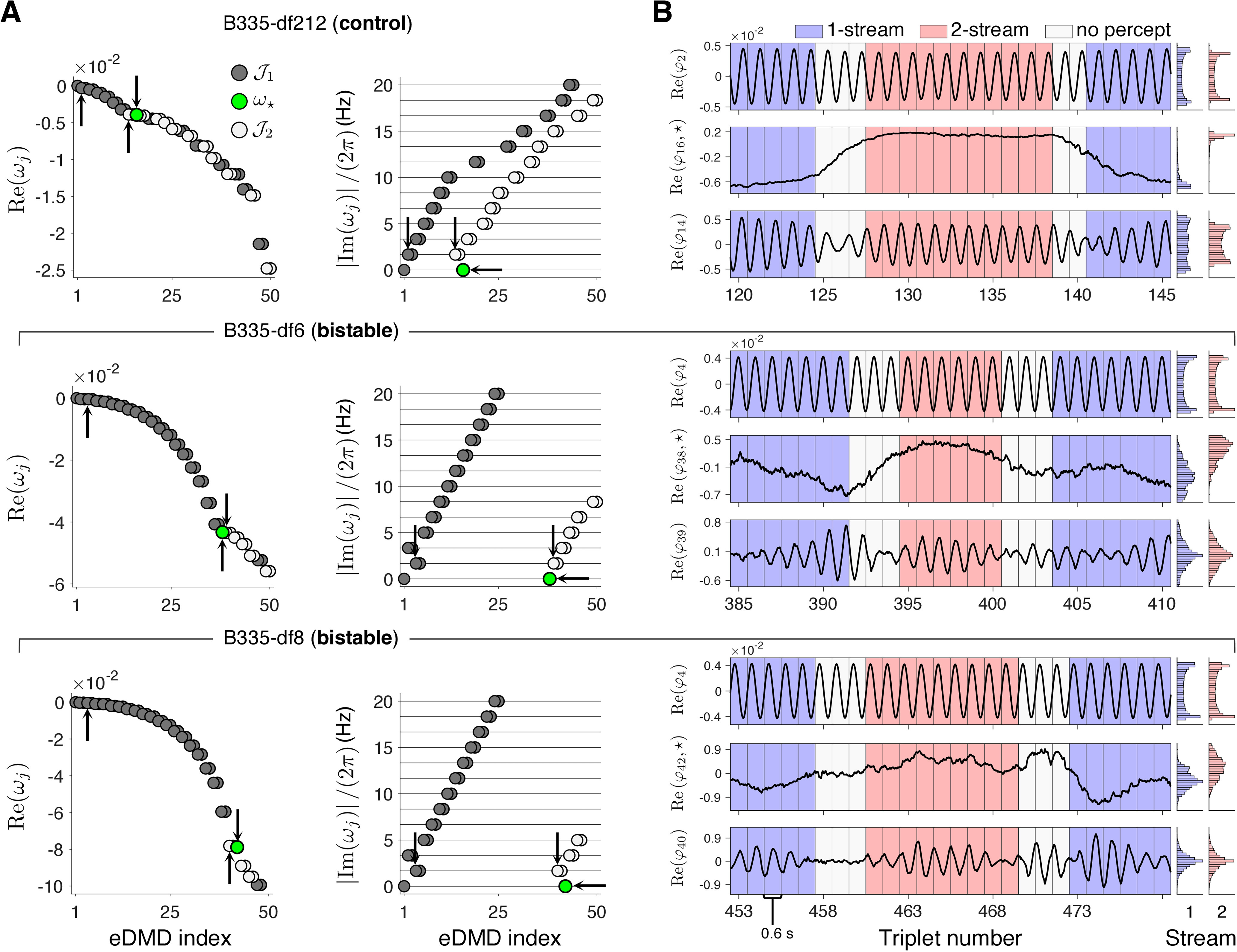
Koopman eigenvalues and eigenfunctions organize into two branches. ***A***, Koopman eigenvalues for subject B335 and df2-12-control (top), *df*6-bistable (middle), *df*8-bistable (bottom). Eigenvalues are indexed according to decreasing real part (left). Imaginary components (right), normalized to have units in Hz, occur at integer multiples of the triplet presentation rate 1.67 Hz, indicated by light gray horizontal lines (only frequencies up 20 Hz are shown). Note the emergence of two eigenvalue branches, $J_{1}$ (dark circles) and $J_{2}$ (light circles). Complex eigenvalues occur in conjugate pairs. Arrows indicate eigenvalues that correspond to eigenfunctions plotted in panel ***B***. ***B***, Real part components of Koopman eigenfunctions for B335 df2-12 control (top), *df*6-bistable (middle), *df*8-bistable (bottom), plotted over select triplets. For each stimulus block, three graphs are shown: one eigenfunction from $J_{1}$ and an eigenfunction from $J_{2}$, both associated with frequency 1.67 Hz (see arrows in ***A***) and the eigenfunction $\varphi_{⋆}$ of eigenvalue $\omega_{⋆}$ (green in ***A***). Background is shaded according to subject-reported perception (one-stream in blue; two-stream in red). On the right, histograms over all sampled time points for each percept-type have bar heights normalized so that the total area for each percept sums to one. Permutation test applied independently to $\varphi_{⋆}$ (indexed 16, 38, 42, respectively) indicated significant differences between means for one-stream versus two-stream perception (*p* < 1.0 × 10^−4^ all three blocks; N=10,000 permutations).

The Koopman eigenvalues $\omega_{j}$, derived with the feature extraction algorithm in Figure 2, were either real (only two) or occurred in complex conjugate pairs (see Fig. 3*A* for eigenvalues from B335). Note that we define $\omega_{j}$ by “exponential eigenvalues,” transforming the discrete Koopman eigenvalues into their continuous-time equivalents; see Materials and Methods. For a complex number $\omega_{j}$, its real and imaginary components encode the decay rate and oscillatory frequency of the corresponding spatial mode $v_{j}$. By convention, the eigenvalues were ordered by decreasing real part. The imaginary components of the eigenvalues, when normalized to have units in Hz, aligned at approximate integer multiples of the triplet presentation rate 1.67Hz (one triplet per 600ms). We identified two collections of eigenvalues exhibiting such an organization for each subject and block *df*. We refer to these collections as branches and collected the indices for branch one and branch two into the indexed sets $J_{1}$ and $J_{2}$, respectively. In short, eigenvalues associated with the harmonics of 1.67Hz appeared in either one or two complex conjugated pairs. For each such harmonic frequency, 1.67Hz, 3.33Hz, 5Hz, 6.67Hz, and so on, we placed the pair of eigenvalues with negative real part closest to zero (i.e., those smallest in magnitude) in $J_{1}$; then we assigned the pair of eigenvalues with larger negative real part, if they existed, in $J_{2}$ (Fig. 3*A*). Thus, from a linear dynamical system perspective, the modes associated with eigenvalues in $J_{1}$ persist over longer time scales than the corresponding modes in $J_{2}$.

The accompanying Koopman eigenfunctions $\varphi_{j}$ represented timeseries neural features throughout the streaming task. The temporal trace of the eigenfunctions associated with each branch differed when compared over the entire 5-min stimulus block. Figure 3*B* illustrates prototypical differences observed between eigenfunctions from $J_{1}$ and $J_{2}$. The first branch contained a constant leading eigenfunction corresponding to the zero eigenvalue (data not shown). The remaining eigenfunctions in $J_{1}$ were oscillatory functions that resembled true sine and cosine harmonics with minimal fluctuations. The second branch contained a purely real eigenfunction that exhibited slow modulations or transitions closely aligned with changes in perception. We denote this eigenfunction and its corresponding eigenvalue by $\varphi_{⋆}$ and $\omega_{⋆}$. (We studied them, for all subjects, in section A slowly-evolving feature encodes the perceptual states, below) The remaining complex eigenfunctions in $J_{2}$ were oscillatory in nature, although they bore less resemblance to pure sine and cosine functions than those from $J_{1}$. The eigenfunctions in $J_{2}$ showed fluctuations and modulations, which were closely entrained to the slow transitions observed in $\varphi_{⋆}$, and thus were similarly aligned with changes in perception (Fig. 3*B*; also discussed below, $J_{2}$*-*features show phase changes aligned with switches in perception).

The eigenvalue organization on branches $J_{1}$ and $J_{2}$, and their associated timeseries eigenfunctions, revealed two distinct aspects of the neural activity. Branch $J_{1}$ identified a prolonged stable periodic process that encoded the *ABA*– triplet repetition rate and its harmonics. Thus, we called $J_{1}$ the “stimulus-encoding” component of the neural signal. Branch $J_{2}$ comprised modes that were frequency-analog to $J_{1}$ but with larger decay rates (Fig. 3*A*, light vs dark circles). They defined an oscillatory process with a relatively faster-changing envelope, found to be entrained to the perceptual states (see next sections). Thus, we called $J_{2}$ the “perception-encoding” component of the neural activity. The branches $J_{1}$ and $J_{2}$ indicated a superposition of two internal brain processes operating on different timescales. To identify task-specific signatures within branches $J_{1}$ and $J_{2}$, we set to examine the properties of timeseries $\varphi_{j}$.

### A slowly-evolving feature encodes the perceptual states

**Figure 4.**
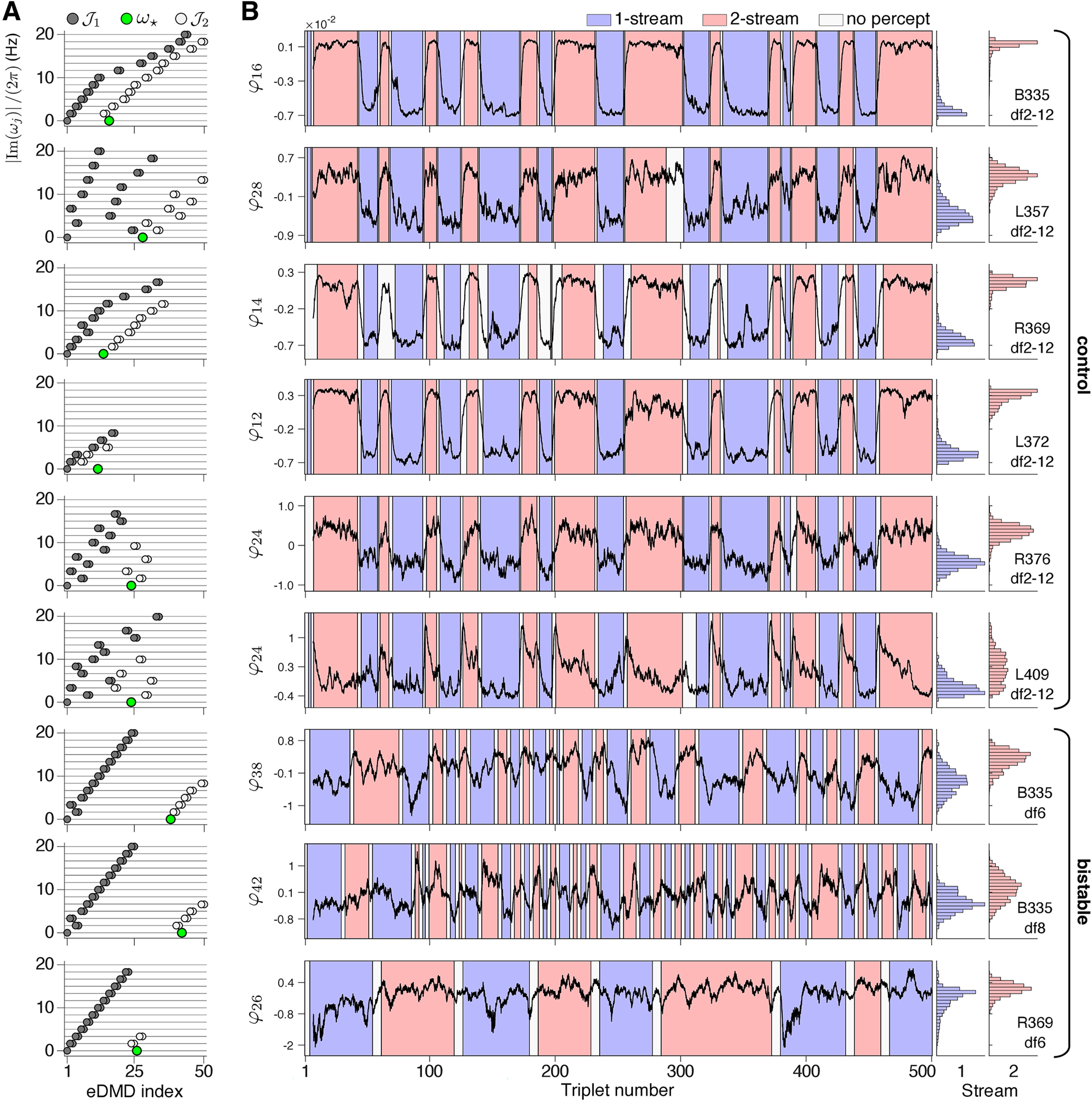
Dynamics of the slowly-evolving feature $\varphi_{⋆}$ on branch $J_{2}$, shown for the entire stimulus duration. ***A***, Sign-free imaginary parts of Koopman eigenvalues from control blocks (top six panels) and bistable blocks (bottom three panels), normalized to have units in Hz. Horizontal lines indicate integer multiples of the triplet presentation rate, 1.67 Hz. Eigenvalue branches $J_{1}$ and $J_{2}$ emerge in all experimental blocks. A complete list of $J_{1}$-eigenvalues includes the following additional harmonics (data not shown): 21.67 Hz for B335, df2-12; 21.67–33.33 Hz for B335, *df*6, *df*8; and 21.67–26.67, 30, 33.33, 36.67 Hz for L357, df2-12. ***B***, Temporal trace for $\varphi_{⋆}$, the real eigenfunction corresponding to the real eigenvalue $\omega_{⋆}$ (green in panel ***A***). Background is shaded according to subject-reported perception (one-stream blue; two-stream red). Triplets preceding button presses were labeled “no-percept” (neutral) to account for individual subject reaction times. Histograms over all sampled time points for each percept-type are shown; bar heights are normalized so that the total area for each percept sums to one. Permutation test applied to $\varphi_{⋆}$ for each subject indicated significant differences between means for one-stream versus two-stream perception (*p* < 1.0 × 10^−4^ in control blocks and in B335 bistable blocks; *p* = 0.0053 in R369 *df*6; *N* = 10,000 permutations).

The eigenfunction $\varphi_{★}$ from $J_{2}$ registered a slow latent variable that transitioned between two attracting-like states (Fig. 4); it emerged in all streaming blocks, with different index in the $\left\{\varphi_{j}\right\}$ sequence. Note that both $\varphi_{⋆}$ and $-\varphi_{⋆}$ were eigenfunctions corresponding to the real eigenvalue $\omega_{⋆}$. By convention, for illustrative purposes, we chose $\varphi_{⋆}$ in the pair above to be the eigenfunction with median calculated across the two-stream percept larger than the median over the one-stream percept (e.g., $\varphi_{⋆}$ as plotted in Figs. 3*B*, 4*B*).

The eigenfunction $\varphi_{⋆}$ differed from the remaining extracted features in that: (1) it was purely real, and thus not entrained to a fixed frequency (the only other real eigenfunction was the constant $\varphi_{1}$); and (2) its dynamics resembled a variable transitioning between two slowly drifting attractor states. The alternations between two relatively steady values of time-series $\varphi_{⋆}$ were aligned with reported changes in perception (Fig. 4*B*). We performed a permutation of triplet labels to test for a difference in the means of $\varphi_{⋆}$ computed across the one-stream and two-stream percepts (see Materials and Methods). We found the difference in percept-related means of $\varphi_{⋆}$ significant at the α=0.01 level. The Monte Carlo *p*-value estimates in all control blocks satisfied $p<1.0\times 10^{-4}$. The *p*-values in the bistable blocks were $p<1.0\times 10^{-4}$ for subject B335 (*df*6 and *df*8) and p=0.0053 for subject R369 *df*6. To quantify the overlap between the distributions of values $\varphi_{⋆}$ split by perceptual grouping (Fig. 4*B*), we calculated the Kullback–Leibler divergence (KLdiv; see function relativeEntropy in MATLAB). Briefly, the amount of shared information between two probability distributions is given by the nonnegative scalar quantity KLdiv. While KLdiv is zero for two identical distributions, it becomes larger when separation between distributions increases (Joyce, 2011). For the histograms shown in Figure 4*B*, we found KLdiv of 33.86, 15.68, 74.02, 47.41, 14.92, and 3.96, with a mean of 31.64, for the control blocks of B335, L357, R369, L372, R376, L409, respectively. We found KLdiv 4.46, 1.75, and 2.58, with a mean of 2.93, for the bistable blocks B335, *df*6, *df*8, and R369, *df*6. The KLdiv values indicated a stronger separation between almost-invariant states identified by $\varphi_{⋆}$ in the control blocks (when perception alternations were more salient because of overlap with stimulus change) than in the bistable blocks (the true bistable conditions).

### $J_{2}$-features show phase changes aligned with switches in perception

**Figure 5.**
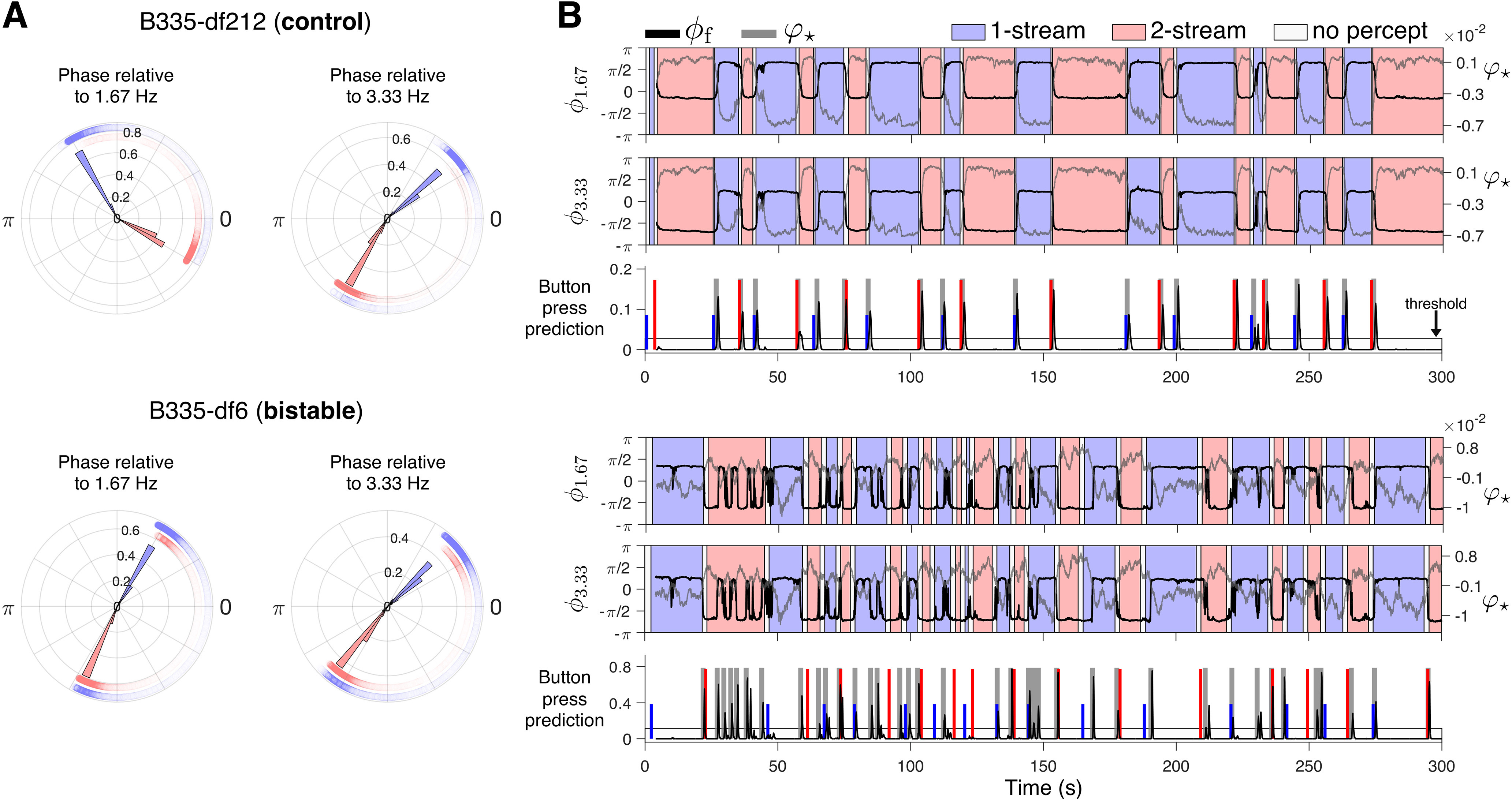
Phase analysis for eigenfunctions from branch $J_{2}$. ***A***, Circular histograms of relative instantaneous phase for B335 df2-12-control (top) and *df*6-bistable (bottom) stimuli, colored according to the subject-reported perception. The instantaneous phase $\phi_{f}$ was calculated with respect to frequency *f* of 1.67 Hz (left) and 3.33 Hz (right; see Eq. 6 in Materials and Methods). It is the phase of eigenfunctions $\varphi_{j}$ indexed with *j* = 14 and 17, respectively, at df2-12, and *j* = 39, 41 at *df*6 in Figure 3*A*. Histogram bins are normalized so that the bin radii for each percept type (one-stream blue; two-stream red) sum to one. Scatter points correspond to phase calculated at individual time points. Marker transparency is scaled according to the density of the phase distribution along −π to π. ***B***, Predicted button presses. Instantaneous phase $\phi_{f}$, in black, is calculated either with respect to frequency 1.67 Hz (top) or to 3.33 Hz (middle). Eigenfunction $\varphi_{⋆}$ is shown in gray. Background is shaded according to subject-reported perception. Button press predictions (bottom) were derived from $\phi_{\text{1}.\text{67}}$ according to Equation 7, in Materials and Methods. Temporal windows containing a predicted button press (gray boxes) are drawn when the predictor variable crosses a threshold (horizontal line). The width of the box was determined by subject reaction-time in response to control stimuli. Short blue (tall red) lines indicate subject-reported switches into one-stream (two-stream) percept.

The emergence of $\varphi_{⋆}$ corresponded to an aforementioned separation of time-scales. We found that the remaining features $\varphi_{j}$ on branch $J_{2}$ were entrained to the modulations observed in $\varphi_{⋆}$. They exhibited phase shifts aligned with the state-transitions of $\varphi_{⋆}$. Namely, as in Figures 3*A* and 4*A*, each eigenfunction $\varphi_{j}$ on branch $J_{2}$ corresponded to a complex eigenvalue $\omega_{j}$ whose imaginary component encoded a frequency, *f*, multiple of 1.67Hz. We computed the instantaneous phase shift $\phi_{j,f}$ of feature $\varphi_{j}$ relative to frequency *f* (see Materials and Methods, Eq. 6) and drew its circular histograms over timepoints separated according to subject-reported percepts. Exemplar histograms and 5-min duration time plots of two instantaneous phases, $\phi_{\text{1}.\text{67}}$, $\phi_{\text{3}.\text{33}}$ are shown in Figure 5. The phases $\phi_{f}$ alternated between two slowly drifting attractor-like states in a manner similar to, but with less fluctuations than, the dynamics of eigenfunction $\varphi_{⋆}$. The phase transitions were also temporally aligned with the steady-state transitions observed for $\varphi_{⋆}$ and, consequently, aligned with reported changes in perception.

### The phase of the leading oscillator in branch $J_{2}$ defines a predictor for perceptual switches

**Figure 6.**
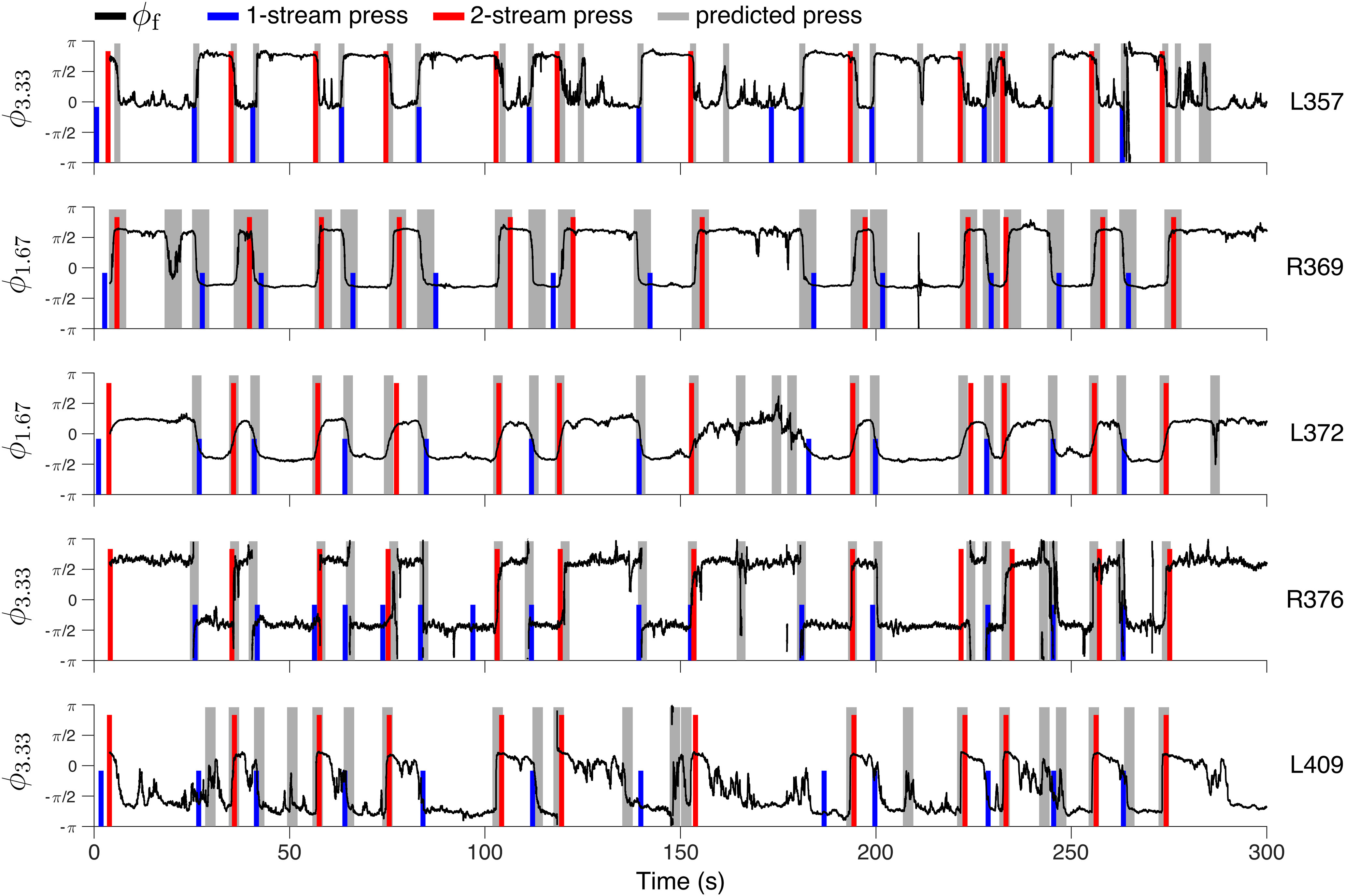
Predicting subject button presses with extracted neural features. Button press predictions are shown for the control blocks of five subjects. In each example, feature $\varphi_{j}$ was chosen from branch $J_{2}$ to correspond to eigenvalue $\omega_{j}$ with frequency *f* (1.67 or 3.33 Hz) as indicated by the vertical axis label, e.g., Figure 4*A*, light circles, for reference. Changes in the instantaneous phase $\phi_{f}$ (black line) of $\varphi_{j}$ determined candidate time windows (gray boxes) for predicted button presses. Phase angles of π and −π are congruent, so transitions through π to −π (or vice versa) appear as jump discontinuities along the vertical axis. The width of the gray box varies between subjects to account for individual differences in button-press reaction time. Subject-reported button press times are marked by colored lines for the onset of one-stream (in blue) and two-stream (in red) percepts. For the prediction criteria, see text.

As opposed to $\varphi_{⋆}$, all frequency-locked instantaneous phases $\phi_{f}$ of $J_{2}$ eigenfunctions took comparable values across subjects and blocks (always in the interval −π to π). They rendered a generic neural measure that encoded, with reasonable accuracy, the timing of the button presses. We selected the instantaneous phase $\phi_{f}$ of the first eigenfunction on branch $J_{2}$ associated with a positive frequency, and used it to identify changes in perception. Depending on subject and block, $\phi_{f}$ was either $\phi_{\text{1}.\text{67}}$ or $\phi_{\text{3}.\text{33}}$ (Figs. 3*A*, 4*A*). At any time point $t_{k}$, we compared the difference between the average phase $\phi_{f;\text{0}.\text{6s}}(t_{k})$, calculated over a preceding temporal span equal to the length of one triplet, with the average phase $\phi_{f;\text{1}.\text{2s}}(t_{k})$, over a span equal to the length of two triplets. The comparison was implemented through a nonlinear predictor variable difference $p_{\phi_{f}}(t_{k})$ that mapped small phase differences toward zero and large phase differences toward one (see Materials and Methods, Eq. 7; also Fig. 5*B*). The difference in average phases was small for the majority of timepoints, which kept $p_{\phi_{f}}(t_{k})$ near zero. The predictor's large deviations from zero prescribed candidate time points for when the button presses might have occurred. The predictor variable was tracked forward in time and monitored for threshold-crossings. Each crossing established a short temporal window containing a predicted button press (Fig. 5*B*, gray boxes). Then these windows were assessed for their alignment with the subject-reported perceptual switches (Fig. 5*B*, blue and red vertical lines). For details about the calculation, see Materials and Methods. Behavioral predictions based on variable $p_{\phi_{f}}$ were found to be reliable. Figure 5*B* shows predicted button-presses and their alignment with the recorded perceptual switching data in df2-12 and *df*6 blocks for subject B335. Button press predictions calculated for the control block of all other subjects are shown in Figure 6.

**Figure 7.**
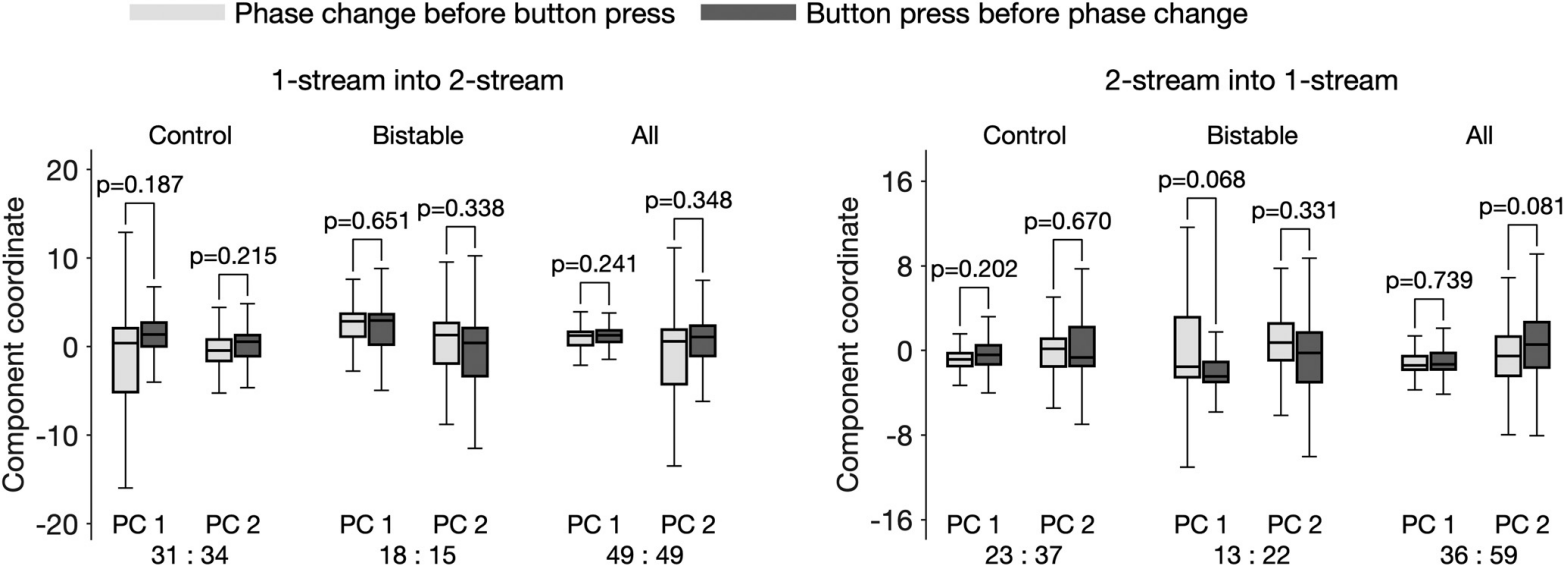
Predicted perceptual switches are not modulated by the subject's motor response. A statistical test was employed to examine whether the algorithm-derived features that correlate with perception reflect neural activity associated with the pressing of buttons. All 1.2-s segments immediately preceding the predicted times for perceptual change were extracted from feature $\varphi_{⋆}$ (Figs. 4, 5; also the definition of predictor $p_{\phi_{f}}$), then *z*-scored and projected onto their first and second principle components. The values were split in two groups, per ratio pb:bp, with pb, the number of events in which the phase-shift-based prediction occurred before the subject's button press (boxplots in light-gray), and bp, the number of events in which the button press appeared before the phase-shift (in dark-gray). Boxplot whiskers extend from 0.25 and 0.75 quantiles to ±1.5 times the interquartile range, respectively. The ratios pb:bp (e.g., 31:34) were shown for each respective case. The analyses were performed separately for transitions from one-stream to two-stream (left panel) and transitions from two-stream to one-stream (right panel), and for the control blocks, the bistable blocks, as well as all blocks together. No statistical significant difference was found (Wilcoxon rank test, *p*-values > 0.05).

To test whether a button press (“observed” value) was more likely to either precede or succeed a predicted switch (“expected” value), based on changes in $\phi_{f}$, we performed a $\chi^{2}$ test with one degree of freedom and *N* samples. From *N* = 126 button presses associated with phase changes in the control blocks (Figs. 5*B*, 6), there were bp = 71 instances of a button press preceding a marked phase change and pb = 55 instances of a phase change preceding a reported button press: $\chi^{2}(1,126)$ = 2.03, *p* = 0.154, not statistically significant. We also performed the analysis over the bistable blocks (bp = 37, pb = 31) and all blocks together (bp = 108, pb = 86) and obtained $\chi^{2}(1,68)$ = 0.53, *p* = 0.467, respectively $\chi^{2}(1,194)$ = 2.49, *p* = 0.114, not statistically significant. The majority of button presses occurring before the predicted phase-shifts (78 out of 108) were found in subjects B335 and L357 with reactions times much shorter than the 1.2s time window used in the definition of the predictor $p_{\phi_{f}}$ (RTs of 0.65, 0.36s). These cases may be explained by the inherent constraints of the proposed feature-extraction algorithm and, possibly, by the subjects' unreliability in pressing the buttons. We investigated here the alternative hypothesis that the feature-derived phase-shifts were a button press artifact, reflecting neural modulation by the motor response. We tested whether changes in the slowly-evolving rhythm $\varphi_{⋆}$ (Fig. 4) associated with predicted perceptual switches occurring before button presses (pb) were indistinguishable from those associated with predictions occurring after button presses (bp). The 1.2s intervals immediately preceding phase shifts were extracted from the $\varphi_{⋆}$ feature. Each 1.2s sample was standardized to have mean zero and variance one to bring the extracted data to a similar scale. Then, the samples were grouped according to transitions from one-stream into two-stream and two-stream into one-stream and projected onto their first and second principal components (Fig. 7). Comparisons over the pb:bp groups, calculated separately for the control blocks, the bistable blocks, and then for all blocks together, showed no significant statistical difference (Wilcoxon rank test, p>0.05; total pb = 85, bp = 108; one early predicted switch in the control block of R369 had to be excluded because of lack of sufficient appended signal history). Similar results were obtained when applying the statistical tests to longer time-intervals preceding the switches, up to 4s history, to account for the delays used in the eDMD part of the algorithm (data not shown).

### Triplet-based mean-LFPs are well approximated by $J_{1}\cup J_{2}$-features

Recall that the feature extraction algorithm processed timeseries $y^{\left(c\right)}(t)$ recorded from all contacts *c* in HGPM, and produced a finite collection of Koopman eigenvalues, modes, and eigenfunctions ${\left\{(\omega_{j},v_{j},\varphi_{j})\right\}}_{j=1}^{N}$ as output (Fig. 2). Then, the projection of $y^{\left(c\right)}(t)$ on the finite-dimensional feature space was a linear combination of time-varying Koopman eigenfunctions $\varphi_{j}$, with time-independent coefficients $v_{j}(c)$,

(3) $$y^{\left(c\right)}(t)\approx \sum_{j=1}^{N}v_{j}(c)\varphi_{j}(t).$$

**Figure 8.**
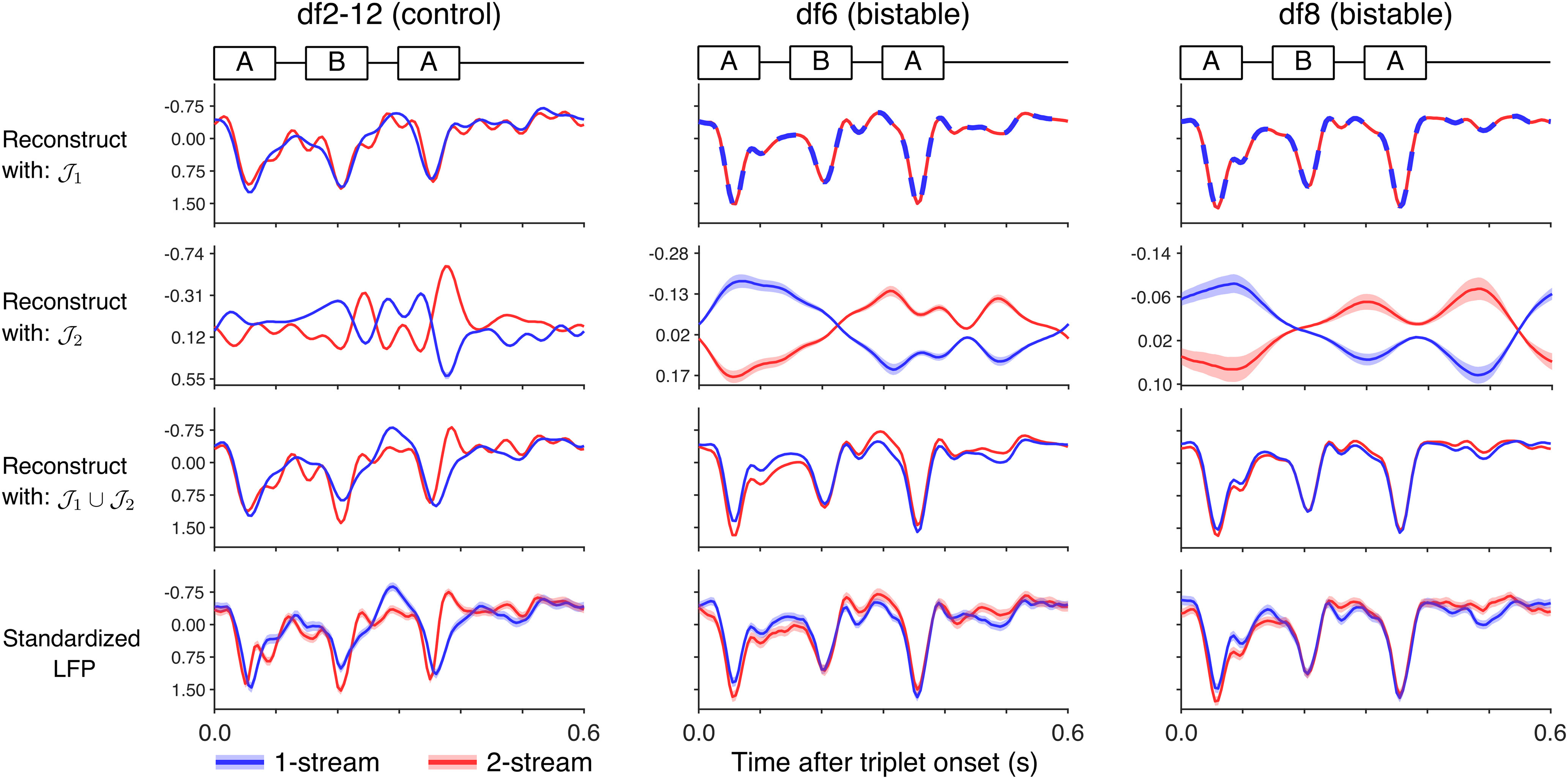
Reconstruction of triplet-based mean-LFPs with extracted features. Eigenfunctions from branches $J_{1}$, $J_{2}$ and the union $J_{1}\cup J_{2}$ were used to construct approximations of LFP recordings, according to Equations 3 and 4. Triplet-based epoch averages of these reconstructions (rows 1–3) are shown for HGPM contact #138 in subject B335, for different stimulus blocks: df2-12 (left column), *df*6 (middle), *df*8 (right). For comparison purpose, the standardized mean-LFPs over triplet-based epochs are also included (row 4). In all panels, the averages were taken over triplet-epochs labeled as one-stream (in blue) and two-stream (in red), according to subject's reported perception (same vertical axis scale in all panels but those for $J_{2}$). 95% CI (SEM) ribbons are drawn (barely visible in most plots, because of very narrow CIs ranges). Branch $J_{1}$-reconstructions preserved the profile of the response to *ABA*– triplets. The apparent triplet structure was no longer preserved in branch $J_{2}$-reconstructions. Reconstructions with $J_{1}\cup J_{2}$ produced profiles similar to the mean-LFPs per percept type.

While the eigenfunctions $\varphi_{j}$ encoded the time dynamics of ECoG recordings, the modes $v_{j}$ provided a spatial map across the contacts in HGPM. Thus, at a fixed contact *c*, the approximation of signal $y^{\left(c\right)}$ employed the same coefficients $v_{j}(c)$ for all times *t*. Each coefficient $v_{j}(c)$ in Equation 3 represented the strength of the relative contribution of $\varphi_{j}$, a temporal component at a particular frequency encoded by $\omega_{j}$, to the reconstruction of ECoG data. To assess what intrinsic dynamical properties of the ECoG signal were expressed along $J_{1}$, $J_{2}$, we split Equation 3 into two terms: $y^{\left(c\right)}$= $Y_{1}^{\left(c\right)}$+$Y_{2}^{\left(c\right)}$ with

(4) $$Y_{1}^{\left(c\right)}=\sum_{j\in J_{1}}v_{j}(c)\varphi_{j},\;Y_{2}^{\left(c\right)}=\sum_{j\in J_{2}}v_{j}(c)\varphi_{j}$$

calculated separately for each branch. Then we computed triplet-based reconstruction profiles by averaging components $Y_{1}^{\left(c\right)}$, $Y_{2}^{\left(c\right)}$ and their sum in Equation 3, over each percept-type (Fig. 8, upper 1–3 rows; mean taken over all triplet-based epochs labeled as either one-stream or two-stream). We compared these quantities with the triplet-based mean-LFPs of the true signal (Fig. 8, 4th row).

We found the average differences between perceptual streams to be limited along branch $J_{1}$ (in fact, differences existed only in the df2-12-control block). On the contrary, $J_{2}$-based reconstructions differed in average between perceptual states. This result was consistent with the properties observed for features on the two branches: eigenfunctions $\varphi_{j}$ in $J_{1}$ exhibited limited deviations from true sine and cosine functions during the 5-min block duration (Fig. 3*B*, upper panel), while eigenfunctions in $J_{2}$ modulated with perception, although on a scale smaller in magnitude (Fig. 3*B*, lower panel; also, Fig. 5). The $J_{1}$-features collectively encoded the stable neural response to the driving stimulus, the sequence of *ABA*– triplets. The $J_{2}$-features collectively encoded the transient dynamics correlated with perception. Thus, $Y_{1}^{\left(c\right)}$ defined the stimulus-encoding component of the LFP recording, while $Y_{2}^{\left(c\right)}$ represented the perception-encoding component.

Similarities between triplet-averaged reconstructions with $J_{1}$-features, computed separately over one-stream and two-stream percepts (Fig. 8, row 1), were estimated with Pearson's correlation coefficient *r* (see Materials and Methods). We found them to be highly correlated, with a median *r* = 0.9996 for reconstructions in bistable blocks (confidence interval 95%CI = [0.9988, 0.9999]) and median *r* = 0.9411 for reconstructions in control blocks (95%CI = [0.9277, 0.9647]). In contrast, the triplet-averaged reconstructions with $J_{2}$-features (Fig. 8, row 2) were highly anticorrelated (in the median, *r* = −0.9846, 95%CI = [−0.9978,−0.9767] in bistable blocks, and *r* = −0.9530, 95%CI = [−0.9650,−0.5179] in control blocks). The mean-LFP $J_{1}$-based profiles over one-stream and two-streams were more strongly correlated for the reconstructions in bistable blocks when the stimuli were unchanging, than in df2-12-control blocks when percept-related differences were confounded with stimulus differences themselves (*r* significantly larger in bistable blocks; one-sided Wilcoxon rank-sum test, $p<1.0\times 10^{-14}$). Likewise, the mean-LFP $J_{2}$-based profiles over one-stream and two-streams were more strongly anti-correlated in bistable rather than control blocks (one-sided Wilcoxon rank-sum test, $p<1.0\times 10^{-6}$).

Next, we assessed the accuracy of the triplet-averaged overall approximation from Equation 3. We calculated the coefficient of determination $R^{2}$ for the feature-based reconstruction mean (Fig. 8, row 3) against the standardized mean LFP (Fig. 8, row 4) separately for each contact, stimulus block, and percept type (N=53 total contact recordings). We found a median $R^{2}$ value of 0.93 for one-stream (first/third quantile: 0.88/0.98) and median $R^{2}$ of 0.95 for two-stream (first/third quantile: 0.89/0.98), in support of a good fit.

### Low-dimensional manifolds extracted from HGPM recordings have a similar geometric structure across group data

**Figure 9.**
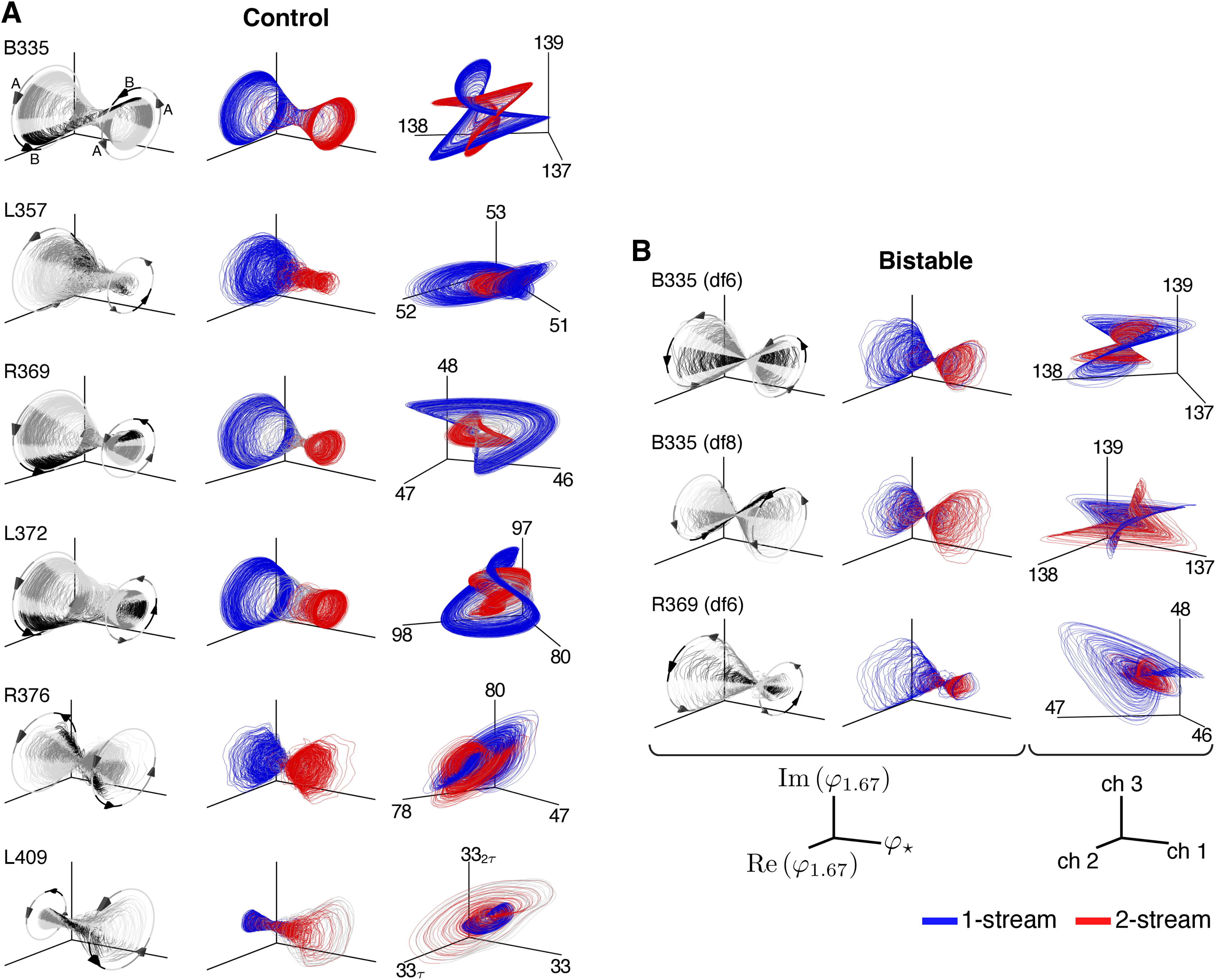
Visualization of low-dimensional neural dynamics extracted from HGPM recordings. ***A***, Three-dimensional projection of the dynamics in df2-12-control blocks of six subjects. Left column, $\varphi_{⋆}$ plotted against the real and imaginary components of the $J_{2}$-eigenfunctions associated with frequency 1.67 Hz. Trajectory is shaded according to triplet *ABA*– composition: *A* tone (dark gray); *B* tone (black); silence (light gray). Arrows and circles indicate direction of rotation for the trajectory and delineate the repetitive *ABA*– stimulus structure. Middle column, Same trajectories from the left column but colored according to subject-reported perception (one-stream, blue; two-stream, red). Time points corresponding to the neutral percept (near button presses) are colored in gray. Right column, Reconstruction of select ECoG contacts in HGPM using only $\varphi_{⋆}$ and eigenfunctions in $J_{2}$ with frequency components 1.67 and 3.33 Hz (see Eq. 4). Points are colored according to reported perception and follow the same convention as the middle column. Subject L409 had a single HGPM contact, so two coordinates delayed by τ = 50 ms were appended for visualizing dynamics in three-dimensions. ***B***, Projection of the dynamics in bistable blocks. Plots follow same convention as in panel ***A***.

For each subject and experimental block, ECoG recordings from all HGPM contacts were introduced as input to the feature-extraction algorithm (Fig. 2). The output was the Koopman collection $\left\{(\omega_{j},v_{j},\varphi_{j})\right\}$ that we analyzed in previous sections. The extracted neural features $\varphi_{j}$ provided a basis for a low-dimensional space on which the 5-min-long timeseries recorded at contact *c* were projected (see Eq. 3). To compare the geometric structures of the Koopman-based spaces across all nine experimental blocks, we plotted the trajectories defined by the first three eigenfunctions in branch $J_{2}$: $\varphi_{⋆}$, $\varphi_{1.67}$, and its complex conjugate ${\bar{\varphi }}_{1.67}$. In real function representation, these trajectories were drawn as curves with coordinates

$$\left(\varphi_{⋆}(t_{k}),\text{Re}\left[\varphi_{1.67}(t_{k})\right],\text{Im}\left[\varphi_{1.67}(t_{k})\right]\right),$$

parameterized by the sampling time $t_{k}$. They formed “double-cone”-like manifolds that were qualitatively similar across subjects and stimulus blocks (Fig. 9, columns 1, 2). Each trajectory showed an oscillation with circular cross-sections parallel to the $\text{Re}[\varphi_{1.67}]\times \text{Im}[\varphi_{1.67}]$ plane, and with the cross-sections' location determined by the values of the slowly-evolving feature $\varphi_{⋆}$.

The double-cone represented an intrinsic invariant structure of the large scale ECoG data, and it was found to be stereotypical across subjects and stimulus type. The oscillations resembled the standard cosine-sine parametrization of the circle with an approximate rotation frequency of 1.67Hz, which is the repetition rate of triplet *ABA*– in the auditory sequence. We found that the timing of each triplet component (the pure tones *A*, *B* and *A*, as well as the 200-ms interval of silence) were encoded at distinct locations on the circle, aligned along the three-dimensional embedding. Trajectories shifted in phase when they drifted to the opposite side of the cone along the $\varphi_{⋆}$-axis (Fig. 9*A*,*B*, left column). Moreover, the double-cone formed a “perceptual manifold,” consisting of two almost-invariant sets corresponding to each percept type (Fig. 9*A*,*B*, middle column: one-stream in blue, two-stream in red). The two-sets intrinsic structure of the underlying manifold was common across subjects.

Low-dimensional dynamics of select HGPM contacts were visualized with projections onto eigenfunctions from branch $J_{2}$. At each contact *c* we considered $Y_{2}^{\left(c\right)}$ from Equation 4 but restricted the summation to the first five terms (the space generated by $\varphi_{⋆}$, $\varphi_{1.67}$, ${\bar{\varphi }}_{1.67}$, $\varphi_{3.33}$, ${\bar{\varphi }}_{3.33}$; see Fig. 4*A*). The dynamics derived at three different HGPM contacts for each subject but L409 are shown in Figure 9*A*,*B*, right column. L409 had only one HGPM contact so we used temporal lags to visualize its three-dimensional neural dynamics. Although the shape of the underlying manifold varied among experimental blocks, its two-attractor-like structure linked to perception was preserved.

## Discussion

We investigated dynamic attributes and underlying geometric structures of neural correlates of auditory streaming of triplets, in minutes-long nonstationary ECoG recordings from human core auditory cortex. These datasets were previously studied by Curtu et al. (2019) with univariate and multivariate statistics, and have revealed significant differences in averaged evoked potentials associated with one-stream and two-stream percepts. Analyses by Curtu et al. (2019) and other studies of auditory streaming (Gutschalk et al., 2005; Cusack, 2005; Kondo and Kashino, 2007, 2009; Hill et al., 2011; Higgins et al., 2020), examined triplet-locked epochs and their percept-related means. Our paper presents a novel analysis of triplet streaming data that exploits their temporal dependencies under prolonged stimulus presentations. The algorithm combines emerging methods for dimensionality reduction, manifold learning, and dynamic discovery. We report a collection of neuronal features that encode changes in auditory bistable perception in their frequency components and instantaneous phases. The extracted features organized in two subsets, $J_{1}$, $J_{2}$, corresponding to stimulus encoding and perception encoding respectively. The dynamics of a slowly fluctuating rhythm $\varphi_{⋆}$ characterized prolonged steady-state percepts. The phase of the leading oscillator in $J_{2}$ was found to be a reliable predictor for the perceptual switches. Low-dimensional projections of the dynamics of neural data revealed a two-attractor-like geometric structure invariant among subjects and stimulus blocks.

In auditory streaming of triplets, alternations between percepts were induced by either stimulus modifications (Sussman et al., 1999; Fishman et al., 2001; Micheyl et al., 2005; Snyder et al., 2006; Pressnitzer et al., 2008; also control blocks df2-12 in our study) or by stimulus bistability (Gutschalk et al., 2005; Hill et al., 2012; Cusack, 2005; Curtu et al., 2019; also bistable blocks *df*6, *df*8). Differences in neural correlates to one-stream and two-stream were identified in MEG (Gutschalk et al., 2005; Billig et al., 2018; Sanders et al., 2018), EEG (Hill et al., 2012; Higgins et al., 2020), fMRI (Cusack, 2005; Kondo and Kashino, 2009; Hill et al., 2011), and ECoG (Curtu et al., 2019) recordings. When changes in low-level acoustic stimulus properties altered perception, it has proven difficult to distinguish stimulus-driven neural signatures from perception-only driven activity. Very few attempts were successful in dissociating such effects (Hill et al., 2012). Our proposed feature-extraction algorithm (Fig. 2) provided a robust solution to the stimulus versus perception separation challenge. The eigenfunctions from the decomposition $J_{1}\cup J_{2}$ (Figs. 3*A*, 4*A*) exhibited distinct properties: those in $J_{1}$ showed percept-independent dynamics and produced a standard profile for the encoding of the *ABA*– triplet repetition; those in $J_{2}$ underwent steady-state phase transitions aligned with changes in perception and produced LFP reconstruction profiles with marked differences between perceptual states (Fig. 8). The reconstructions with $J_{1}$-features of mean triplet-based LFPs supported the hypothesis that $J_{1}$ is associated with the stimulus encoding, as profiles were aligned across percept types for the bistable stimuli but deviated for the control stimuli that included triplets with different *A*-tones. The features on $J_{2}$ indicated a representation of an internal percept, although stimulus-related effects could not be entirely excluded. Statistically significant differences between percept-split distributions of $\varphi_{⋆}$ values were identified in both control and bistable blocks, but they were larger in the control conditions (Figs. 3*B*, 4*B*). The stronger separation of distributions observed in df2-12 blocks possibly reflected the saliency of the perceptual switches, which were more prominent because of overlap with the stimulus change. Another explanation, that the distinctive low-level acoustic components of *df*2, *df*12 sequences significantly modulated the quasi-states of $\varphi_{⋆}$ seemed improbable, given that stimulus attributes evolved on a much faster timescale than $\varphi_{⋆}$, and that each contact in HGPM sampled large populations of neurons with wide tone-frequency selectivity.

Phase modulation of cortical oscillations as a potential mechanism for neural encoding of auditory streaming has been studied in rodents (Noda et al., 2013). Under stimulus modifications used as proxies for the perceptual states, differences in “percept”-related phase coherences were reported in the γ frequency-band. In contrast, our results showed that perceptual phase modulations (for features in $J_{2}$) were tightly linked to the harmonics of the triplet presentation rate rather than existing in a specific frequency-band.

Features in $J_{1}$, $J_{2}$ and their qualitative dynamics, were invariant across subjects and stimulus types. Branch $J_{2}$ contained a real eigenfunction $\varphi_{⋆}$ whose slowly-evolving dynamics correlated with perception. The other eigenfunctions were ordered by the decay rates of their associated eigenvalues and had frequencies near 1.67Hz harmonics. The phase of $J_{2}$ leading oscillator was used to predict individual timings of the button presses. Some individual differences existed, however. For certain subjects the algorithm identified 1.67Hz as the frequency of the persistent oscillation in $J_{2}$. This was true for B335 and R369, regardless of stimulus type, and L372. For other subjects (L357, R376, L409) this frequency was 3.33Hz (Figs. 3*A*, 4*A*). We hypothesize that the leading frequency in $J_{2}$ gives an indication of the strategy each subject used when identifying the two-stream percept. While some experiments with auditory triplet streaming asked the participants to attend certain aspects of the stimulus (e.g., to exclusively follow either the *A* tone, or the *B* tone, in the two-stream percept; Gutschalk et al., 2005; Snyder et al., 2006; Thompson et al., 2011; Billig et al., 2018), we provided no such instruction. The subjects may have unconsciously directed their attention to the *A* tone (presented every 0.3s in the triplet sequence, at 3.33Hz rate) or to the *B* tone (presented every 0.6s, at 1.67Hz rate). This is a testable hypothesis that could be addressed in future studies of triplet streaming by appropriately adjusting the set of instructions.

Like classic signal processing methods, our feature-extraction algorithm identified fundamental frequencies in recordings and derived linear decompositions in frequency-selected components. There are, however, key differences between these approaches. First, Figure 2-algorithm processed data from all HGPM contacts together and produced a common collection of features for the decomposition of individual LFPs. The frequencies (harmonics of 1.67Hz) and associated time-varying elements arose naturally from the data and were computed simultaneously as Koopman eigenvalues and eigenfunctions. This contrasts canonical methods that treat recordings independently, extract fundamental frequencies with power spectrum calculations, and then partition recordings into frequency-specific modulated timeseries with techniques like the fast-Fourier transform (FFT). Identifying a common space for HGPM neural activity proved essential for the discovery of geometric and dynamic structures shared across group data. Second, as opposed to basic sine and cosine FFT-functions, the Koopman eigenfunctions were nonstationary throughout the 5-min stimulus. They were oscillatory, “Fourier-like,” yet adaptive to the intrinsic LFP dynamics during the perceptual task. Third, discovery of the slow rhythm $\varphi_{⋆}$ by conventional Fourier methods would have been impossible. The ECoG data were high pass filtered at 1.5Hz (see Materials and Methods; preprocessing), having all additive low-frequency components removed. However, low-frequency multiplicative signals remained as they modulated signals at higher frequencies (e.g., the phase and amplitude of $J_{2}$-eigenfunctions were modulated by $\varphi_{⋆}$). These features were identified and extracted through Koopman projections.

Computational models proposed several neural mechanisms for perceptual bistability. These included oscillatory and noise-driven attractor dynamics (Laing and Chow, 2002; Moreno-Bote et al., 2007; Curtu et al., 2008; Rankin et al., 2015), evidence accumulation (Nguyen et al., 2020), predictive coding (Denham and Winkler, 2006), and probabilistic rule-based classification processes (Barniv and Nelken, 2015; Steele et al., 2015). To our knowledge, our study is the first to: (1) report neural evidence for within-trial ongoing competing percepts and (2) report neural activity compatible with the dynamics of models for bistable perception. We extracted a feature of the neural activity in core auditory cortex, $\varphi_{⋆}$, that correlated with single-trial behavioral responses. The instantaneous phase of an entire ensemble of oscillators (in branch $J_{2}$) did the same. These latent variables exhibited alternating dynamics similar to those simulated by attractor-based models.

Oscillatory attractor dynamics rely on three key computational principles: bistability between two stimuli-induced attractors (typically, two stable steady states), processes for accumulation then recovery for drifting activity between attractors, and noise to ensure trial-by-trial variability. The first two principles underlie a deterministic periodic trajectory that alternates between attractors. The noise introduces stochasticity in the switching times and creates more realistic, irregular percept durations. The model can be augmented by coupling it to a chaotic system to account for external factors that influence perception (e.g., attention, medication). Figure 2-algorithm approximated the quasi-periodic solution of such attractor-like flow through features in $J_{1}\cup J_{2}$. Diffusion maps parametrize paths between competing states in stochastic systems governed by a double-well potential (Nadler et al., 2006). In those examples, the first nonconstant diffusion map eigenvector described the alternation between attracting regions. Our results identified feature $\varphi_{⋆}$ deeper in the Koopman spectrum, near the start of branch $J_{2}$. A possible explanation is that the two hypothesized attractors underlying the ECoG data were not equilibria but periodic cycles themselves, generated by the repetitive *ABA*– triplet sequence. The algorithm likely captured the time-dependent structure of the stimulus before extracting the intrinsic system dynamics.

Bridging modeling with large-scale neural recordings has recently received scientific interest because of new methodologies like manifold learning and Koopman decomposition. Theoretical results found that DMD alone (Tu et al., 2014) was not well-suited for identifying slow or transient dynamics as seen in perceptual switching. This shortcoming was addressed by eDMD (Williams et al., 2015) together with time-delay coordinates (Takens, 1981) and diffusion maps (Berry et al., 2013). The Koopman operator was then shown to accurately describe quasi-periodic solutions of oscillatory systems with “weak” chaotic components (Giannakis, 2019), although it seemed unable to track chaotic dynamics per se (Mezić, 2005, 2013; Giannakis, 2019). We combined these methods into a single algorithm to extract neuronal features that captured single-trial behavioral responses in auditory streaming of triplets. Our results do not suggest that bistable perception might be resolved in primary auditory area HGPM. Rather they show that, under certain circumstances, one could make predictions of ongoing perceptual alternations by simply recording from HGPM.

## Materials and Methods

### Participants

Six neurosurgical patients treated for pharmaco-resistant epilepsy participated in the streaming task: four males and two females, identified here as B335, L357, R369, L372, R376, and L409 (age range 29–47; median age 33 years). The subjects listened to sequences of repeated triplets of tones and reported their perception, either one-stream or two-stream, by pressing a button. Electrocorticographic (ECoG) data from multicontact depth electrodes and subdural electrode arrays and subject behavioral responses were recorded simultaneously. Research procedures were approved by The University of Iowa Institutional Review Board and the National Institutes of Health. Participation in the task was voluntary, and each patient had the option to cease participation at any time without disruption in their clinical treatment. These datasets were previously published by Curtu et al. (2019).

### Auditory stimuli and bistable perception

The *ABA*– stimulus structure followed the description by Curtu et al. (2019). Tones *A* and *B* lasted 100ms, with 10ms raised cosine onset and offset ramps. Brief 50ms intervals of silence followed the first *A* and *B* tone, and 200ms of silence followed the second *A* tone, with a total duration of 600ms per triplet. Participants listened to a sequence of 500 triplets (5 min in duration), called a stimulus block.

For all participants, except R369, and all stimulus blocks, the *B* tone frequency was fixed at $f_{B}$=1000Hz (1250Hz for R369). The frequency of the *A* tone varied between $f_{A}$= 1122, 1414, 1587, and 2000 Hz (for R369: 1403, 1768, 1984, and 2500 Hz), corresponding to semitone differences of *df* = 2, 6, 8, and 12, respectively. Three stimulus blocks were considered: df2-12, *df*6, and *df*8. The df2-12 block was defined by a repetition of triplets that alternated between *df* = 2 and *df* = 12 semitone differences (12 durations for each of *df*2 and *df*12 ranging between 5–28 and 9–45 triplets, respectively). The timing of the onset and offset of *df*2 and *df*12 epochs was identical across subjects. We called df2-12 the *control* block since it elicited stable percepts, with listeners generally reporting the *df*2 semitone difference as one-stream (integrated ABA−ABA−...) and the *df*12 semitone difference as two-stream percept (segregated *A*–*A*–*A*–*A*–... and –*B*–*B*–...). The stimulus in the *df*6 and *df*8 blocks did not change throughout the behavioral task. The frequency of the *A* and *B* tones remained fixed, separated by *df* = 6 semitones (*df* = 8, respectively) in all 500 triplets, but subjects reported spontaneous switches between one-stream and two-stream percepts. We named *df*6 and *df*8 the (perceptually) bistable blocks.

All subjects were instructed to report changes in perception from one-stream into two-stream or two-stream into one-stream by pressing an appropriate button on a response box. Reaction times (RTs) were computed from the latency of their behavioral responses to stimulus changes during the control block (0.65, 0.36, 3.22, 1.24, 1.29, and 1.48s for B335, L357, R369, L372, R376, and L409). To account for individual reaction times, we assigned in our analysis a percept-neutral label (neither one-stream nor two-stream) to a number of triplets immediately preceding each button press: in bistable blocks, two triplets for B335 and six triplets for R369; in control blocks, all triplets following a stimulus change and until the button press was recorded (see below; also Curtu et al., 2019).

### ECoG recordings and data preprocessing

The ECoG data that we analyzed in this paper were a subset of the datasets published by Curtu et al. (2019), and were preprocessed following the same procedure. Briefly, ECoG recordings were obtained simultaneously from multicontact depth electrodes and subdural electrode arrays placed over the temporal, parietal, and frontal lobes. Data were acquired using an RZ2 real-time processer from Tucker-Davis Technologies for subjects B335 and L357 and the Neuralynx ATLAS system for R369, L372, R376, and L409. Depth electrode arrays (8–12 macro contacts, spaced 5mm apart) were stereotactically implanted along the anterolateral-to-posteromedial axis of Heschl's gyrus and provided coverage of core (posteromedial Heschl's gyrus) and noncore (anterolateral Heschl's gyrus) auditory cortex (Fig. 1*C*). Neural signals were also obtained from contacts targeting other noncore auditory areas within the superior temporal plane (planum temporale, planum polare), insula and superior temporal sulcus (using depth electrodes), and from surrounding auditory-related temporoparietal cortex and frontal areas (subdural grid arrays). The ECoG recordings were amplified, filtered (0.7- to 800-Hz bandpass, 12dB octave rolloff), digitized at a sampling rate of 2034.5Hz (Tucker-Davis Technologies) or 2000Hz (Neuralynx), and saved along with the time series of behavioral reports (button presses) for future analyses. The data from each recording site were then downsampled to 1000Hz, denoised using the method proposed by Kovach and Gander (2016), and screened for possible contamination from electrical interference, epileptiform spikes, high-amplitude and slow-wave activity. Finally, the data were bandpass filtered between 1.5 and 70 Hz, with the frequency components in the narrow band 2.2 to 2.7 Hz discarded by temporal and spatial filters. For detailed description of the procedure, see Curtu et al. (2019). Note that we optimized the selection of the parameter *N* in the spatial filter that determines how many components of the singular value decomposition (SVD) of the normalized spatial correlation matrix, taken over all contacts in the narrowly defined frequency band, were to be discarded. Here, the value of *N* was derived independently for each individual subject by applying a previously described criterion (Gavish and Donoho, 2014) to their ECoG data (as opposed to choosing N=30 across all subjects as in Curtu et al., 2019).

### Experimental design and statistical analysis

#### Input to the feature extraction algorithm

Our analyses were performed on a subset of the complete ECoG data set. We retained as input into the proposed algorithm only the ECoG recordings obtained from contacts in posteromedial Heschl's gyrus (HGPM). The number of HGPM contacts, $n_{c}$, per individual subject was six (B335), five (L357), eight (R369), six (L372), seven (R376), and one (L409); (e.g., Fig. 1*C* for B335). Nine perceptual blocks were analyzed independently: the control block df2-12 for all subjects; the bistable block *df*6 of B335 and R369; and bistable block *df*8 of B335 (Figs. 4, 9). At each contact *c*, the local field potential ${\text{LFP}}_{c}(t)$ was normalized along a 3-s sliding window and, for computational efficiency, further downsampled at 200 Hz. Briefly, at each time point $t_{k}$ we standard-normalized ${\text{LFP}}_{c}(t_{k})$ into

$${\left[\text{LFP}\right]}_{k}^{\left(c\right)}=({\text{LFP}}_{c}(t_{k})-\mu_{k}^{\left(c\right)})/\sigma_{k}^{\left(c\right)},$$

where $\mu_{k}^{\left(c\right)}$ and $\sigma_{k}^{\left(c\right)}$ were the mean and standard deviation of contact ${\text{LFP}}_{c}(t)$ computed over the 3-s window centered at time $t_{k}$. (For time-points within 1.5s of stimulus onset and offset, the time window used for standard-normalization partially included brief intervals of time preceding or succeeding the streaming task.). Timeseries ${\left[\text{LFP}\right]}_{k}^{\left(c\right)}$ were smoothed by averaging over a 10ms window centered at each $t_{k}$. Then, the signal was downsampled by taking one time point at every 5ms. The sampling rate of 200Hz is consistent with other dynamic mode decomposition applications [e.g., 200Hz for 20min recordings in B.W. Brunton et al. (2016), 500Hz for 45s in Marrouch et al. (2018), and 100Hz for 1.4s in Cura and Akan (2020)]. These transformed local field potentials (LFPs), denoted by $y_{k}^{\left(c\right)}$, have a reduced number of time samples $t_{k}$, 60,000 (instead of 300,000 or more) and are ready for implementation into the algorithm outlined in Figure 2.

#### Time-delayed coordinates and diffusion maps

The first step in the analysis was to perform the state-space reconstruction and nonlinear dimensionality reduction of HGPM data per experimental block. This was done using the diffusion-mapped delay coordinate (DMDC) method outlined by Berry et al. (2013), with the following variables and hyperparameter selection. We considered $y_{k}^{\left(c\right)}$ as above and constructed the ensemble of ECoG measurements made at time $t_{k}$ for all $n_{c}$ HGPM contacts: $y_{k}=[y_{k}^{\left(1\right)},...,y_{k}^{\left(n_{c}\right)}]\in {\mathbb{R}}^{n_{c}}$. This vector was then augmented to the α-weighted delay coordinates:

(5) $${\tilde{y}}_{k}≜\left[y_{k},e^{-\alpha }y_{k-1},...,e^{-s\alpha }y_{k-s}\right]\in {\mathbb{R}}^{n_{c}(s+1)}.$$

by appending *s*-number of previous measurements. As proposed by Berry et al. (2013), the exponential factor in the delay coordinates was introduced to improve regularity in the embedded coordinates. Berry et al. (2013) advised to choose a small nonzero constant for the decay α to balance information loss (at large α the tail of delay coordinates decays rapidly to zero) with reduced regularization (at α= 0). We set α= 0.001 for all datasets and appended each observation with *s* = 799 past measurements. Thus, a single delay coordinate ${\tilde{y}}_{k}$ contained neural information from a temporal window spanning 4 s before time $t_{k}$ (since (799+1)timepoints×5ms/timepoint=4000ms=4s). This delay length of 4 s prevented alignment with the triplet presentation rate, to avoid stimulus-based periodicities in the neural data, and balanced computational performance with empirical performance across subjects and stimulus blocks.

Note that the first 799 $t_{k}$-samples (representing the measurements made in the 4 s immediately following stimulus onset) were not included in the analyses of the extracted features because there was insufficient history to append to them. Hence, only 59,201 data points $y_{k}$ were embedded as ${\tilde{y}}_{k}$ in ${\mathbb{R}}^{n_{c}(s+1)}$=${\mathbb{R}}^{n_{c}\times 800}$ (equivalent to time spanning 296s instead of 300s, total stimulus duration in each block).

The delay coordinates are hypothesized to encapsulate key dynamical properties of the original data. To generate low-dimensional representations of these dynamics, we introduced them as input into a diffusion map algorithm (Coifman et al., 2005; Coifman and Lafon, 2006). This algorithm performs nonlinear dimensionality reduction by calculating eigenvalues and eigenvectors of a stochastic matrix P derived from a nonlinear kernel. The kernel is rotationally invariant and chosen to emphasize local neighborhoods and geometry intrinsic to the data. Such an emphasis is an alternative to linear directions of maximal variance, as identified in principal component analysis (PCA). Here, we used the Gaussian kernel as a similarity measure between all delay coordinates ${\tilde{y}}_{i}$ and ${\tilde{y}}_{j}$,

$$k_{\text{G}}({\tilde{y}}_{i},{\tilde{y}}_{j})=\exp\left[-\frac{||{\tilde{y}}_{i}-{\tilde{y}}_{j}|{|}^{2}}{\varepsilon^{2}}\right],$$

with ε a data-dependent parameter, and ||⋅|| representing the standard Euclidean norm. Parameter ε provides a notion of locality as the kernel function $k_{\text{G}}$ decays to zero fast when evaluated at points away from ${\tilde{y}}_{i}$, at distance larger than ε. Setting ε too small or too large could lead to ill-posed eigenvalue problems or distort approximations predicted by diffusion map theory (e.g., if ε is too small, the points become isolated; if it is too big, the neighborhoods become too large to be relevant). It is thus beneficial to make ε data dependent. We chose ε to be the average of the $k_{\text{min}}$ = 12 nearest neighbor distances computed for each data point, as described by Berry et al. (2013). Then, the kernel was sparsified by setting points outside of $k_{\text{nn}}$ = 192 neighborhood to be zero; for any fixed point ${\tilde{y}}_{i}$, if ${\tilde{y}}_{j}$ is not among the $k_{\text{nn}}$ near neighbors of ${\tilde{y}}_{i}$, then the kernel takes negligible values, and we set $k_{\text{G}}({\tilde{y}}_{i},{\tilde{y}}_{j})\equiv 0$. In close analogy to PCA, we computed leading eigenvalues and eigenvectors $\left\{(\lambda_{j},\psi_{j})\right\}$ of the matrix P, $P\psi_{j}=\lambda_{j}\psi_{j}$. The eigenvectors from diffusion maps approximate heat diffusion along the underlying manifold M and provide timeseries of Fourier-like functions adapted to dynamics along M, ordered according to decreasing eigenvalues (Coifman and Lafon, 2006; Berry et al., 2013; Giannakis, 2019). In this way, the eigenvectors from diffusion maps are a natural fit to preserve low-dimensional dynamic and geometric structures revealed with delay coordinates. We view each $\psi_{j}$ as a timeseries by identifying the $k^{\text{th}}$ component of $\psi_{j}$ with $\psi_{j}(k)=\psi_{j}(x(t_{k}))$, the ${}_{k}$-time sample of some hidden state of the brain dynamics that generated the HGPM data. The leading *N*-eigenvectors identified as output of the DMDC algorithm would form a dictionary of functions in the extended dynamic mode decomposition (eDMD) algorithm proposed by Williams et al. (2015).

### Extended dynamic mode decomposition

The eDMD algorithm requires the specification of a dictionary of basis-like functions to approximate the eigenfunctions of the Koopman operator associated with the system's dynamics as linear combinations of dictionary elements. We developed a procedure to select the input dictionary for the eDMD algorithm. By computing the fast-Fourier transform (FFT) of previously identified DMDC eigenvectors $\psi_{j}$, we observed a frequency-based structure: DMDC leading eigenvectors contained frequency components centered near harmonics of the triplet presentation rate (every 600ms), with components of 1.67Hz, 3.33Hz, 5Hz, and so on. The leading vectors were followed by (and in some cases interleaved with) another set of vectors, which also contained spectral components near the aforementioned frequencies. However, these vectors included more frequency components as well as modulations not observed in the leading eigenvectors. They also exhibited a reduction in spectral power compared with the first collection. A third set of vectors were characterized by further reduction in spectral power and had no clear organization according to frequency.

We took the starting index of the third subset of vectors as a hard threshold for function selection: the eDMD dictionary was defined by all DMDC eigenvectors before this index. Note that while the exact cutoff index varied, the qualitative organization of the eDMD dictionary was similar across subjects.

With the selected DMDC functions, we performed the eDMD algorithm as described by Williams et al. (2015) to obtain approximations to Koopman eigenvalues, modes, and eigenfunctions: ${\left\{(\omega_{j},v_{j},\varphi_{j})\right\}}_{j=1}^{N}$. The real and imaginary parts of eigenvalues $\omega_{j}$ encode a decay rate and an intrinsic frequency associated with eigenfunction $\varphi_{j}$. The frequency is expressed in cycles per second (or Hz) by dividing the imaginary part of $\omega_{j}$ by 2π. We used the decay rate and intrinsic frequency of $\omega_{j}$ to organize $\varphi_{j}$ into two distinct branches, named here $J_{1}$ and $J_{2}$ (see Fig. 3*A*). Eigenfunctions $\varphi_{j}$ encode key temporal features of recordings in HGPM over the 5-min stimulus block (less the first 4 s because of delay-coordinate constraints), with the value $\varphi_{j}(k)$ representing the sampling at time $t_{k}$. These eigenfunctions are neural features extracted by sampling along the manifold M underlying the dynamics of the data. Finally, each eigenmode $v_{j}\in {\mathbb{R}}^{n_{c}}$ encodes the weights $v_{j}(c)$ of the contribution of neural feature $\varphi_{j}$ to the spatial pattern of activity at all *c* recording sites.

In this paper, we identified changes in the temporal dynamics of neural features $\varphi_{j}$ that correlated with perceptual changes in the auditory streaming task (Fig. 4). We then used the weights from $v_{j}$ to reconstruct triplet-averaged approximations of the ECoG recordings from HGPM (Fig. 8).

### Almost-invariant dynamical regions

Our analysis showed that trajectories along the manifold M evolved in two distinct separated regions for long periods of time, with fast transitions between them. We adopted the terminology from Froyland and Padberg (2009) and called these regions “almost-invariant” (Fig. 9, blue vs red regions). While such subsets of M were not dynamically invariant (trajectories eventually left them, often in a chaotic or probabilistic way), they were consistent with the almost-invariant sets. In accord with the definition by Froyland and Padberg (2009), a trajectory originating in either almost-invariant attracting set of M was unlikely to leave the region for some nontrivial amount of time; then the wandering time in the complementary set, before the return, was also nontrivial.

### The eigenvalues of the Koopman operator

The values $\omega_{j}$ from the extended dynamic mode decomposition are the “exponential” Koopman eigenvalues. We obtained them by transforming the eigenvalues $\lambda_{j}$ of the discrete-time Koopman operator into their continuous-time counterparts according to formula $\omega_{j}=\ln(\lambda_{j})/\Delta t$. Here, $\Delta t=t_{k+1}-t_{k}$ represented the time discretization of the ECoG measurements $y_{k}$ and was equal to 5ms (see above, Input to the feature extraction algorithm). Mathematically, $\lambda_{j}^{k}=e^{\omega_{j}(\Delta t)k}=e^{\omega_{j}t_{k}}$, which can be written as: $e^{\omega_{j}t_{k}}=e^{Re(\omega_{j})\;t_{k}}e^{i\;Im(\omega_{j})\;t_{k}}$. For the discrete-time Koopman operator, the decaying modes are associated with values $\lambda_{j}$ inside the unitary circle: $|\lambda_{j}|<1$. In the continuous-time exponential eigenvalues interpretation, the condition for the decaying modes translates into $\omega_{j}$ belonging to the negative half-plane: $Re(\omega_{j})<0$. Thus, $Re(\omega_{j})$ defines the decay rate of the corresponding spatial mode $v_{j}$, while the imaginary part $Im(\omega_{j})$ encodes the mode's oscillatory frequency.

### Permutation test

To study the statistical significance of the correspondence between eigenfunction $\varphi_{⋆}$ and perception we compared the mean of $\varphi_{⋆}$ over triplet-locked epochs from the subject-reported one-stream and two-stream percepts ($\varphi_{⋆}$ is described in Results; see also Figs. 3, 4).

The null hypothesis of the permutation test (to be rejected) states that there is no difference in the mean of $\varphi_{⋆}$ over the two percepts. Here, we implemented a permutation test in which the triplet labels (one-stream vs two-stream) were shuffled, while maintaining the number of reported switches per stimulus block. Therefore, each permutation test randomly assigned the same number of perception switches within the 500-triplet stimulus as reported by the subject. The placement of switches was constrained by the subject's reaction time, RT: new “percepts” were created by gluing together a number of same-label-triplets to cover a time window larger than RT. The first switch was always placed at the beginning of the stimulus after accounting for RT. Once all switches were in place, the first percept was randomly assigned to either one-stream or two-stream category. All triplets from the beginning to the second randomly placed switch took the label of that percept. Then the perception was changed from one-stream into two-stream or two-stream into one-stream, and all triplets up to the third switch were associated with the new percept type. The alternating process continued until all triplets were assigned a one-stream or two-stream label. As with the subject-reported behavioral data, triplets preceding each switch received a neutral label to account for RT. This labeling process produced random perception assignments to triplets consistent with perception reports for each subject. It prevented unlikely outcomes like markedly short or long perceptual states (which could be produced if triplet labels were purely randomly assigned), with respect to individual differences in switching rates.

For each permutation of labels, we calculated the difference in means of $\varphi_{⋆}$ over the numerically generated one-stream and two-stream percepts, $\mu_{\text{perm}}=\mu_{\text{2s},\text{perm}}-\mu_{\text{1s},\text{perm}}$. This difference was compared with the difference in means for the observed perception, $\mu_{\text{obs}}=\mu_{\text{2s},\text{obs}}-\mu_{\text{1s},\text{obs}}$. We counted how many times, *m*, the absolute value $\left|\mu_{\text{perm}}\right|$ was larger or equal to $\left|\mu_{\text{obs}}\right|$ among the *n* = 10,000 permutations, and derived the Monte Carlo *p*-value estimate p=(m+1)/(n+1). Percept-related differences in mean were considered significant at the α= 0.01 level.

### Instantaneous phase

Any complex number z=a+ib can be written in trigonometric form $z=re^{\text{i}\theta }$ with θ between −π and π defined by the four-quadrant arctangent, θ = atan2(Im[z],Re[z]) = atan2(b/a). Accordingly, for any complex eigenfunction $\varphi_{j}$ obtained by eDMD algorithm, we computed the instantaneous angle

$$\theta_{j}(t_{k})=\text{angle}\left(\varphi_{j}(t_{k})\right)=\text{atan2}\left(\text{Im}[\varphi_{j}(k)],\text{Re}[\varphi_{j}(k)]\right),$$

then defined the trigonometric instantaneous phase shift of $\varphi_{j}$ relative to a frequency *f* in Hz by

(6) $$\phi_{j,f}(t_{k})=\text{angle}\left(e^{\text{i}\left(2\pi ft_{k}-\theta_{j}(t_{k})\right)}\right).$$

Recall that the imaginary components of eDMD-derived eigenvalues $\omega_{j}$ have close correspondence with integer multiples *m* of the triplet presentation rate 1.67Hz. Thus, for each eigenfunction $\varphi_{j}$, we set the frequency *f* to the harmonic f=m×1.67Hz which is the closest to $\text{Im}[\omega_{j}]/(2\pi )$. In particular, if such an eigenfunction were a perfect sine or cosine generated at frequency *f* then the instantaneous phase shift computed according to Equation 6 would be constant throughout the entire 5 min experimental block. In this paper we refer to the instantaneous phase shift $\phi_{j,f}(t_{k})$ relative to a particular frequency *f* in Equation 6 as simply *the instantaneous phase*. In some examples, when we wanted to emphasize the dependence of the phase on the frequency component *f*, as opposed to the eDMD index *j*, we used the short notation $\phi_{f}$ instead of $\phi_{j,f}$ (see Figs. 5, 6).

As a timeseries, the instantaneous phase shift $\phi_{j,f}(t_{k})$ of certain eigenfunctions $\varphi_{j}$ (for indices $j\in J_{2}$) exhibited switching dynamics between two steady-states. Typically, these steady-state angles were nearly antipodal and lied approximately on opposite sides of the unit circle. Then the transient time points alternated from one state to another along the unit circle, passing back and forth either through the 0 angle (as in blocks df2-12, *df*6, *df*8 for B335 and *df*6 for R369; see Fig. 5), or through the opposite angle ±π (blocks df2-12 for subjects L357, R369, L372, R376, L409). For the latter, to account for the periodic boundary conditions on the circle and avoid discontinuities in trajectories crossing ±π, we rotated all instantaneous phases $\phi_{j,f}(t_{k})$ by π radians. As such, we forced the majority of transient points to fluctuate between the two steady-states through angle 0 before calculating the four-quadrant arctangent (an action equivalent to mapping $\varphi_{j}(t_{k})$ into $-\varphi_{j}(t_{k})$), and established comparable timeseries plots across all experimental blocks. These shifts were performed solely for illustrative purposes and had no impact on the analysis.

### Button press prediction

We used the instantaneous phase $\phi_{j,f}$ of a certain eigenfunction ${\hat{\varphi }}_{j}$ from the eDMD algorithm to construct a predictor for behavior. For each experimental block, we selected ${\hat{\varphi }}_{j}$ from branch $J_{2}$ as follows: ${\hat{\varphi }}_{j}$ was the eigenfunction associated with the Koopman eigenvalue ${\hat{\omega }}_{j}$ of the slowest decay rate, $\text{Re}[{\hat{\omega }}_{j}]$, and of positive frequency, $\text{Im}[{\hat{\omega }}_{j}]/(2\pi )$. Since eDMD assigns indices *j* in decreasing order of real part for eigenvalues, and since all complex eigenvalues come in conjugate pairs, ${\hat{\varphi }}_{j}$ is simply the first eigenfunction on branch $J_{2}$ associated with a positive frequency (e.g., $\varphi_{14}$ for B335 df2-12 in Fig. 3). Frequency *f* was selected to be the closest harmonic of 1.67Hz to frequency $\text{Im}[{\hat{\omega }}_{j}]/(2\pi )$. Depending on the experimental block, this was either *f* = 1.67Hz or *f* = 3.33Hz. Next, for simplicity, the selected eigenfunction ${\hat{\varphi }}_{j}$ and its instantaneous phase $\phi_{j,f}$ relative to this frequency *f* were renamed $\varphi_{f}$ and $\phi_{f}$ (e.g., $\phi_{1.67}$, $\phi_{3.33}$ for different blocks in Fig. 6).

We computed $\phi_{f}(t_{k})$ according to Equation 6 then averaged it over two temporal windows immediately preceding time point $t_{k}$, with the time interval spanning one triplet (0.6s in length) and two triplets (1.2s), respectively:

$$\phi_{f;0.6\text{s}}(t_{k})=\underset{t\in [t_{k}-0.6,t_{k}]}{\text{mean}}\phi_{f}(t),\;\phi_{f;1.2\text{s}}(t_{k})=\underset{t\in [t_{k}-1.2,t_{k}]}{\text{mean}}\phi_{f}(t).$$

These were calculated with “CircStat” toolbox for MATLAB (Berens, 2009). The average phases, as opposed to the instantaneous phase, had the advantage of reducing the effects of rapid but unsustained fluctuations in $\phi_{f}$, and allowed for a comparison of phase transition from triplet to triplet along the streaming sequence. To predict the occurrence of the button presses, we constructed a nonlinear function

(7) $$p_{\phi_{f}}(t_{k})={\left[\sin\left(\frac{\phi_{f;0.6\text{s}}(t_{k})-\phi_{f;1.2\text{s}}(t_{k})}{2}\right)\right]}^{2}$$

that tracked the two average phases above and mapped their differences into values between 0 and 1. The nonlinear predictor $p_{\phi_{f}}(t_{k})$ was chosen to emphasize antipodal phase differences (i.e., angle differences close to −π or π) by mapping them into one, and to suppress similar phase differences (i.e., for angle differences near −2π, 0, or 2π) by mapping them close to zero. In other words, persistent differences that lie on nearly opposite sides of the unit circle will map close to one under Equation 7. We referred to the output of Equation 7 as the predictor variable.

The instantaneous phase $\phi_{f}(t_{k})$ was found to be largely stable during the block duration, apart from rapid transitions between steady-states. Thus, the predictor variable maintained a value close to zero at most time points. The predictor variable's rare but large deviations from zero were used to identify candidate timings for predicted button presses. We calculated the mean $\mu_{p}$ and standard deviation $\sigma_{p}$ of the predictor variable over all time points $t_{k}$, and took one standard deviation above the mean as threshold for predictive perception change. Starting with the time at stimulus onset, each time point $t_{k}^{*}$ for which the predictor variable exceeded the threshold, $p_{\phi_{f}}(t_{k}^{*})>\mu_{p}+\sigma_{p}$, marked a predicted switch in perception. We hypothesized that a button press should have occurred inside a time window near $t_{k}^{*}$. We defined this temporal interval $I(t_{k}^{*})$ by $t_{k}^{*}-1.2\leq t\leq t_{k}^{*}+\text{RT}$ (in s), taking into account the triplet repetition rate as well as the subject's RT. To avoid overlap of prediction intervals, we also imposed a refractory period of RT+1.2s following $t_{k}^{*}$ for any new possible “switches.” In the end, all time intervals for predicted button presses, $I(t_{k}^{*})$, detected with the neural predictor $p_{\phi_{f}}$ as above, were verified for their alignment with the behavioral reports (subject-reported perception changes).

### Signal reconstruction

System observations (LFPs) were approximated as linear combinations of the 5 min long Koopman eigenfunctions using the elements in the Koopman modes $v_{j}\in {\mathbb{R}}^{n_{c}}$ as coefficients for individual contacts (see Eq. 3 in Results). The feature extraction algorithm highlighted two distinct branches $J_{1}$, $J_{2}$ of eigenfunction membership. To assess the contributions of each branch, the summation was taken exclusively with eigenfunctions from either $J_{1}$ or $J_{2}$ as in Equation 4.

Triplet-based reconstruction profiles ${\bar{Y}}_{1}^{\left(c\right)}$, ${\bar{Y}}_{2}^{\left(c\right)}$, ${\bar{Y}}^{\left(c\right)}$ were computed by averaging components $Y_{1}^{\left(c\right)}$, $Y_{2}^{\left(c\right)}$ and entire sum $Y_{1}^{\left(c\right)}+Y_{2}^{\left(c\right)}$ over each percept-type (mean taken over all triplet-based epochs labeled as either one-stream or two-stream). These quantities were compared with the triplet-based averages ${\bar{y}}^{\left(c\right)}$ of the original signal.

Similarities between triplet-averaged reconstructions ${\bar{Y}}_{1}^{\left(c\right)}$ computed separately over one-stream and two-stream percepts were estimated with Pearson's correlation coefficient, *r*. These measures were calculated per individual subject, block, and contact, for a total of 53 comparisons. The median *r* values were calculated for the group data in control blocks (median of 33 *r* values obtained from 33 contacts) and for the group data in bistable blocks (median of 20 *r* values from 20 contacts). Then 95% confidence intervals to the median were derived with bias-corrected and accelerated percentile bootstrap sampling (Efron and Tibshirani, 1994; DiCiccio and Efron, 1996), with 10,000 bootstrap samples each. Differences in the median correlation *r* of bistable group data versus control group data were tested for significance with one-sided Wilcoxon rank-sum exact tests at the α=0.01 level of significance. The same procedure quantified similarities/dissimilarities between triplet-averaged reconstructions ${\bar{Y}}_{2}^{\left(c\right)}$ over one-stream and two-stream percepts.

### Assessing the effects of noise on time-delay embeddings

**Figure 10.**
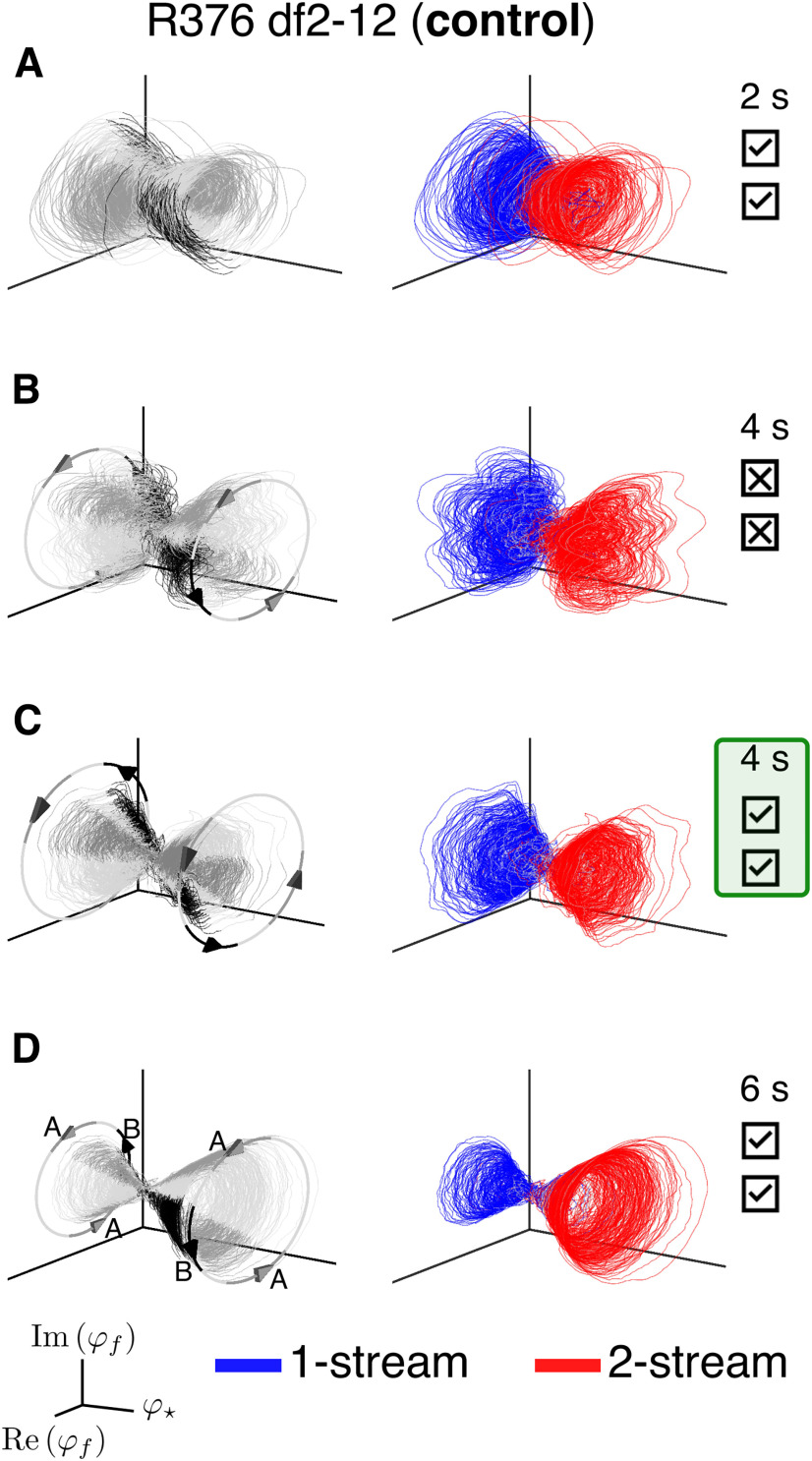
Mitigation of noise effects in low-dimensional embeddings built by the feature selection algorithm. To assess the robustness of the algorithm and it sensitivities to preprocessing, we varied the number of appended delays: (***A***) 2 s, (***B***, ***C***) 4 s, and (***D***) 6 s, respectively, and either included (***A***, ***C***, ***D***) or excluded (***B***) the 3 s sliding window standard-normalization of ECoG data and the 10 ms local averaging (see Materials and Methods for details on hyperparameter selection). Algorithmic results for subject R376, block df2-12, are shown. We plotted along the three axes the eigenfunction $\varphi_{⋆}$ and the real and imaginary parts of the leading oscillator $\varphi_{f}$ from branch $J_{2}$, respectively. ***A***, In the case of 2 s delay, the algorithm identified the lowest frequency of eigenvalues on branch $J_{2}$ at 3.33 Hz (not 1.67 Hz), so $\varphi_{f}$=$\varphi_{3.33}$. Nevertheless, the embeddings plotted along the corresponding eigenfunctions together with $\varphi_{⋆}$ still recovered the one-stream versus two-stream perceptual regions. ***B***, The perceptual region and triplet-tone alignment manifested in the embedding, but the low-amplitude oscillations along the trajectories indicated a contaminating frequency. ***C***, The standardization and averaging attenuated the contaminating frequency found in panel ***B***, acting successfully toward noise reduction. This is the embedding from Figure 9. ***D***, Further increasing the delay improved the control of the noise in the construction of the low-dimensional embedding. In panels ***B–D***, the leading oscillator was $\varphi_{1.67}$.

Time-delay embeddings, particularly those constructed from real data, have been shown to be sensitive to noise. Establishing a methodology for the attenuation of the effects of noise on embeddings is still an open area of research (Ignacio et al., 2019; in topological data analysis) and (Pan and Duraisamy, 2020; in nonlinear dynamical systems). We relied on these references, and on others (see below), to build several components of the feature extraction algorithm that mitigate the impacts of noise on the results. First, we scaled the delayed-coordinates by an exponentially decaying coefficient $e^{-s\alpha }$ to control the temporal history of the signal (see Eq. 5). As shown by Berry et al. (2013), increasing parameter *s* leads to a reduction of the level of noise. This scaling controls the impact of datapoints further away in time from $y_{k}$. Second, we used diffusion maps in conjunction with the delayed coordinates. Reduction techniques like the singular value decomposition (SVD) have been shown to be robust to noise (Gavish and Donoho, 2014). The diffusion map reduction is analog to SVD or the principal component analysis, except that its kernel is nonlinear and is designed to preserve the intrinsic geometry revealed by the time-delayed coordinates. Finally, we aimed to further attenuate noise in the recordings by implementing two additional preprocessing steps: standard-normalizing the local field potentials (LFPs) with a sliding 3s window and time-averaging over a short 10ms window (see above, Input to the feature extraction algorithm). Since many state-space reconstruction results hold in the limit of a large number of delays (Giannakis, 2019), we took a pragmatic approach to assessing the impacts of noise. We studied the effect on the results when we changed the number of delays *s*, and when we did or did not apply the aforementioned preprocessing steps. In our hyperparameter exploration, we focused on three measures: (1) the emergence of the two eigenvalue branches $J_{1}$, $J_{2}$; (2) the identification of eigenfunction $\varphi_{⋆}$ on $J_{2}$ that correlates with behavior; and (3) the properties of the low-dimensional dynamics associated with the auditory streaming task. We found that the feature extraction algorithm preserved the qualitative structure and interpretability of the results in all our parameter combinations. Figure 10 shows the effect on low-dimensional embeddings for subject R376, block df2-12, when we implemented the following four parameter combinations: 2 s time-delays plus temporal-standard normalization of LFPs plus averaging over the 10ms window (Fig. 10*A*), increase delays to 4s but remove the 3s time standardization as well as remove the 10ms window averaging (Fig. 10*B*), 4s delays with standardization and with averaging (Fig. 10*C*; this is the parameter set we used in the manuscript for all subjects), and increase delays to 6s while keeping the standardization and averaging procedures (Fig. 10*D*). To summarize, increasing the number of delays led to reduced noise artifacts in the uncovered features, but also smeared the jumps between near-steady states in $\varphi_{⋆}$, and between the phase transitions, over wider time intervals. In order to balance the reduction of noise in output features with steep transitions in $\varphi_{⋆}$, and to keep a uniform set of hyperparameters across all subjects and stimulus blocks, in Results, we implemented the strategy described in Figure 10*C*.

### Code accessibility

All code, data, and analysis files for this report are available at https://osf.io/7w9qh/.
